# Modeling the mechanobioelectricity of cell clusters

**DOI:** 10.1007/s10237-020-01399-0

**Published:** 2020-11-03

**Authors:** Alessandro Leronni

**Affiliations:** Department of Civil, Environmental, Architectural Engineering and Mathematics (DICATAM), University of Brescia, Via Branze, 43, 25123 Brescia, Italy

**Keywords:** Bioelectricity, Poromechanics, Electro-diffusion, Osmosis, Electro-osmosis, Membrane potential

## Abstract

We propose a continuum finite strain theory for the interplay between the bioelectricity and the poromechanics of a cell cluster. Specifically, we refer to a cluster of closely packed cells, whose mechanics is governed by a polymer network of cytoskeletal filaments joined by anchoring junctions, modeled through compressible hyperelasticity. The cluster is saturated with a solution of water and ions. We account for water and ion transport in the intercellular spaces, between cells through gap junctions, and across cell membranes through aquaporins and ion channels. Water fluxes result from the contributions due to osmosis, electro-osmosis, and water pressure, while ion fluxes encompass electro-diffusive and convective terms. We consider both the cases of permeable and impermeable cluster boundary, the latter simulating the presence of sealing tight junctions. We solve the coupled governing equations for a one-dimensional axisymmetric benchmark through finite elements, thus determining the spatiotemporal evolution of the intracellular and extracellular ion concentrations, setting the membrane potential, and water concentrations, establishing the cluster deformation. When suitably complemented with genetic, biochemical, and growth dynamics, we expect this model to become a useful instrument for investigating specific aspects of developmental mechanobioelectricity.

## Introduction

Recent endeavors have documented that, alongside genetic and biochemical cues, bioelectrical and mechanical signaling is important for development (McCaig et al. 2005; Mammoto and Ingber 2010).

In particular, *bioelectricity* deals with the study of the ion redistribution within a network of non-excitable cells and its environment, modulating the membrane potential (Levin et al. 2017). The latter qualifies as both a key readout and regulator of several developmental processes, such as proliferation and differentiation (Sundelacruz et al. 2009), at the single cell level, and symmetry breaking (Levin et al. 2002), wound healing and regeneration (Levin 2007), and cancer progression (Chernet and Levin 2013), at the tissue level.

In addition to experimentation through ion channels manipulation, deciphering the bioelectrical dynamics requires ad hoc simulators. The *BioElectric Tissue Simulation Engine* (BETSE), a finite volume code proposed by Pietak and Levin (2016), allows one to predict the spatiotemporal evolution of the ion concentrations and membrane potential within a cluster of closely packed cells, in response to a perturbation of the bioelectric state. Later, BETSE has been augmented to consider the interplay between genetic, biochemical, and bioelectrical dynamics, so as to explain aspects of planaria regeneration (Pietak and Levin 2017) and developmental brain damage and rescue in frogs (Pai et al. 2018).

Notably, as argued by Silver and Nelson (2018), bioelectrical and mechanical cues are expected to affect each other. Indeed, on the one hand, the membrane potential and the osmotic pressure are strictly related. On the other hand, several ion channels are mechanosensitive, that is, they respond electrically to changes in the membrane mechanics (Martinac 2004).

Then, Silver et al. (2020) have proved that mechanotransduction may effectively direct the establishment of membrane potential gradients within a tissue. In particular, they show that connexin hemichannels, which are mechanosensitive, preferentially open in the peripheral regions of mammary epithelial tissues, characterized by a higher endogenous mechanical stress, thus leading to local depolarization. This, in turn, is responsible for transcriptional changes that promote cell proliferation.

In order to numerically address the coupling between mechanical and bioelectrical signaling, BETSE has been endowed with a solid mechanics module in Leronni et al. (2020), leading to mecBETSE. Specifically, such a module allows one to compute the cluster deformation due to the osmotic pressure gradients determined by the bioelectrical activity and to account for mechanosensitive channels.

However, mecBETSE is limited to small deformations, thus hampering developmental applications. Moreover, it does not account for the water flow triggered by osmotic pressure gradients, which, according to *poromechanics* frameworks (Coussy 2004), largely employed for cells (Moeendarbary et al. 2013), mechanistically establishes the deformation field.

So as to overcome the previous limits, we propose a continuum finite strain theory coupling the bioelectricity and the poromechanics of cell clusters. In this theory, the bioelectrical response is governed by mass balances for the intracellular and extracellular concentrations of mobile ions and by Gauss laws for the intracellular and extracellular electric potentials. The poromechanical response is determined by mass balances for the intracellular and extracellular concentrations of water molecules and by a momentum balance for the displacement field of the solid network of cytoskeletal filaments and anchoring junctions.

After introducing the object of modeling, that is, the cluster with its main constituents involved in the mechanobioelectrical response, in Sect. 2, we systematically derive the theory in Sect. 3, starting from first principles. In Sect. 4, with reference to a 1D axisymmetric benchmark, we discuss the finite element solution of the proposed model obtained through the commercial software *Comsol Multiphysics*^®^. Finally, in Sect. 5 we draw the conclusions of our study and outline possible future developments.

## Modeling object

**Fig. 1:**
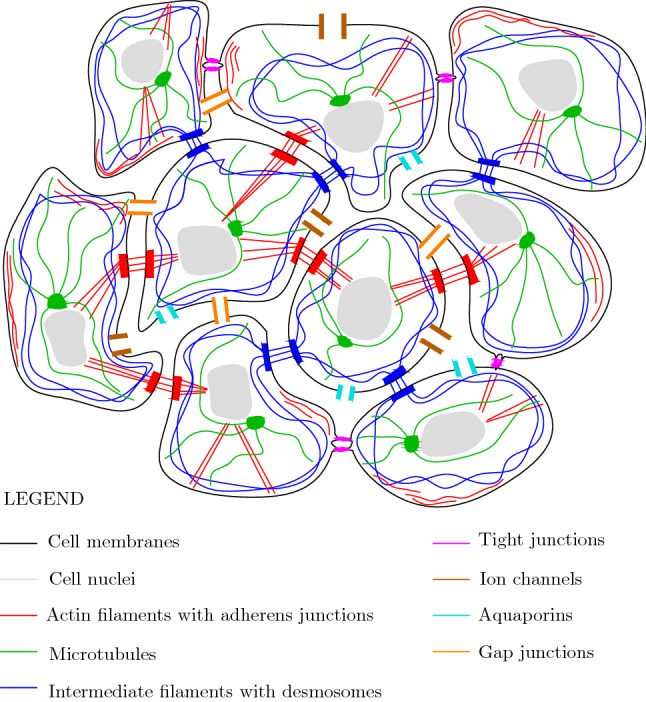
Cell cluster with the main constituents involved in the mechanobioelectrical response

We refer to a cluster of closely packed animal cells, as sketched in Fig. 1. Each cell is endowed with a cytoskeleton of actin filaments, microtubules, and intermediate filaments. Similar to epithelia, actin and intermediate filaments of neighboring cells are mechanically joined by adherens junctions and desmosomes, respectively (Alberts 1983). Importantly, there exists a thin space between cells (Tsukita et al. 2001), constituting a porosity network referred to as the *extracellular* (EC) space, within which water and ions can freely flow. At the cluster boundary, we assume that perfectly sealing tight junctions (TJs) (Tsukita et al. 2001) may be either absent or present, respectively allowing or preventing the water and ion exchange between the EC space and the bath surrounding the cluster. Water and ions can also flow directly between neighboring cells through gap junctions (GJs) (Goodenough and Paul 2009; Gao et al. 2011). We refer to the porosity network formed by the cytoplasm and the GJs as the *intracellular* (IC) space. Finally, the *transmembrane* water and ion transport, that is, the transport between the IC and EC spaces, is allowed by aquaporins (Agre 2006) and ion channels (Hille 1984), respectively. In a cluster of plant cells, anchoring junctions are replaced by rigid cell walls, and plasmodesmata play the role of GJs (Alberts 1983).

## Modeling framework

In the following, we develop a continuum Lagrangian finite strain theory addressing the mechanobioelectricity of the cell cluster described in Sect. 2. By relying on *mixture theory* (Ateshian 2007), we assume that the solid network and the IC and EC spaces coexist within the same material point, such that transmembrane fluxes should be regarded as *local* fluxes.

### Balance equations

The momentum balance, to be solved for the displacement field ${\mathbf {u}}$, describes the mechanics of the solid network of cytoskeletal filaments and anchoring junctions. Under the quasi-static approximation, and in the absence of bulk loads, it reduces to the mechanical equilibrium

1 $$\begin{aligned} {\mathrm {Div}}\,{\mathbf {P}}={\mathbf {0}}\,, \end{aligned}$$

where ${\mathrm {Div}}$ is the material divergence and ${\mathbf {P}}$ is the *nominal* stress tensor, such that $({\mathrm {Div}}\,{\mathbf {P}})_i=\partial P_{iJ}/\partial X_J$, in which ${\mathbf {X}}$ denotes the material position vector and the small and capital case subscripts indicate the spatial and material coordinates, respectively.

The mass balances for the IC and EC water concentrations read

2a $$\begin{aligned}&\varPhi _0{\dot{C}}_{\mathrm {w}}+{\mathrm {Div}}\,{\mathbf {J}}_{\mathrm {w}}=-J_{\mathrm {w}}^{\mathrm {m}}A^{\mathrm {c}}/V^{\mathrm {c}}\,, \end{aligned}$$

2b $$\begin{aligned}&\varPhi _0^{{\mathrm {e}}}{\dot{C}}_{\mathrm {w}}^{\mathrm {e}}+{\mathrm {Div}}\,{\mathbf {J}}_{\mathrm {w}}^{\mathrm {e}}=J_{\mathrm {w}}^{\mathrm {m}}A^{\mathrm {c}}/V^{\mathrm {c}}\,, \end{aligned}$$

where $\varPhi _0$ and $\varPhi _0^{\mathrm {e}}$ are the initial IC and EC porosities, $C_{\mathrm {w}}$ and $C_{\mathrm {w}}^{\mathrm {e}}$ are the IC and EC molar concentrations of water per unit *reference* volume of the IC and EC spaces, ${\mathbf {J}}_{\mathrm {w}}$ and ${\mathbf {J}}_{\mathrm {w}}^{\mathrm {e}}$ are the IC and EC nominal molar fluxes of water, $J_{\mathrm {w}}^{\mathrm {m}}$ is the transmembrane nominal molar flux of water, positive if water moves from the IC to the EC space, $A^{\mathrm {c}}$ is the reference cell membrane area, and $V^{\mathrm {c}}$ is the reference cell volume. The symbol $\dot{}$ indicates time derivative, that is, ${\dot{C}}_{\mathrm {w}}=\partial C_{\mathrm {w}}/\partial t$. Since the cluster is constituted by closely packed cells, $\varPhi _0^{\mathrm {e}}\ll \varPhi _0$. The terms on the right-hand sides are self-balancing, that is, a source of water for the IC space is a sink for the EC space, or vice versa.

Similarly, the mass balances for the IC and EC concentrations of mobile ion ${\mathrm {i}}$ read

3a $$\begin{aligned}&\varPhi _0{\dot{C}}_{\mathrm {i}}+{\mathrm {Div}}\,{\mathbf {J}}_{\mathrm {i}}=-J_{\mathrm {i}}^{\mathrm {m}}A^{\mathrm {c}}/V^{\mathrm {c}}\,, \end{aligned}$$

3b $$\begin{aligned}&\varPhi _0^{\mathrm {e}}{\dot{C}}_{\mathrm {i}}^{\mathrm {e}}+{\mathrm {Div}}\,{\mathbf {J}}_{\mathrm {i}}^{\mathrm {e}}=J_{\mathrm {i}}^{\mathrm {m}}A^{\mathrm {c}}/V^{\mathrm {c}}\,, \end{aligned}$$

where $C_{\mathrm {i}}$ and $C_{\mathrm {i}}^{\mathrm {e}}$ are the IC and EC molar concentrations of ion ${\mathrm {i}}$ per unit reference volume of the IC and EC spaces, ${\mathbf {J}}_{\mathrm {i}}$ and ${\mathbf {J}}_{\mathrm {i}}^{\mathrm {e}}$ are the IC and EC nominal molar fluxes of ion ${\mathrm {i}}$, and $J_{\mathrm {i}}^{\mathrm {m}}$ is the transmembrane nominal molar flux of ion ${\mathrm {i}}$.

For the sake of clarity, ${\mathbf {J}}_{\mathrm {w}}$ and ${\mathbf {J}}_{\mathrm {i}}$, ${\mathbf {J}}_{\mathrm {w}}^{\mathrm {e}}$ and ${\mathbf {J}}_{\mathrm {i}}^{\mathrm {e}}$, and $J_{\mathrm {w}}^{\mathrm {m}}$ and $J_{\mathrm {i}}^{\mathrm {m}}$ represent the water and ion fluxes between cells through GJs, in the interconnected intercellular spaces, and across cell membranes through aquaporins and ion channels, respectively.

Finally, the Gauss laws for the IC and EC electric potentials $\psi$ and $\psi ^{\mathrm {e}}$ read

4a $$\begin{aligned}&{\mathrm {Div}}\,{\mathbf {D}}=\varPhi _0F\sum _{\mathrm {i}}z_{\mathrm {i}}C_{\mathrm {i}}\,, \end{aligned}$$

4b $$\begin{aligned}&{\mathrm {Div}}\,{\mathbf {D}}^{\mathrm {e}}=\varPhi _0^{\mathrm {e}}F\sum _{\mathrm {i}}z_{\mathrm {i}}C_\mathrm {i}^{\mathrm {e}}\,, \end{aligned}$$

where ${\mathbf {D}}$ and ${\mathbf {D}}^{\mathrm {e}}$ are the IC and EC nominal electric displacements, *F* is the Faraday constant, and $z_{\mathrm {i}}$ is the valency of ion ${\mathrm {i}}$. The terms on the right-hand sides represent the IC and EC nominal free charges, and account for both mobile and fixed ions.

### Boundary and initial conditions

In our benchmark, we consider that the cluster is traction-free, that is, we supplement Eq. (1) with the boundary condition

5 $$\begin{aligned} {\mathbf {P}}{\mathbf {N}}={\mathbf {0}}\,, \end{aligned}$$

where ${\mathbf {N}}$ is the outward unit normal to the reference boundary.

The initial conditions for the mass balances (2) and (3) read

6a $$\begin{aligned}&C_{\mathrm {w}}=C_{\mathrm {w}}^0\,,\quad C_{\mathrm {w}}^{\mathrm {e}}=C_{\mathrm {w}}^{\mathrm {e,0}}\,, \end{aligned}$$

6b $$\begin{aligned}&C_{\mathrm {i}}=C_{\mathrm {i}}^0\,,\quad C_{\mathrm {i}}^{\mathrm {e}}=C_{\mathrm {i}}^{\mathrm {e,0}}\,. \end{aligned}$$

We assume that water and ions can be exchanged with the bath surrounding the cluster through the EC space only, provided that TJs are absent. Therefore, we equip Eqs. (2a) and (3a) with the boundary conditions

7a $$\begin{aligned}&{\mathbf {J}}_{\mathrm {w}}\cdot {\mathbf {N}}=0\,, \end{aligned}$$

7b $$\begin{aligned}&{\mathbf {J}}_{\mathrm {i}}\cdot {\mathbf {N}}=0\,, \end{aligned}$$

where the symbol $\cdot$ denotes the inner product, such that ${\mathbf {J}}\cdot {\mathbf {N}}=J_IN_I$.

As for Eqs. (2b) and (3b), in the absence of TJs we impose chemical equilibrium at the boundary, that is,

8a $$\begin{aligned}&\mu _{\mathrm {w}}^\mathrm {e}={\bar{\mu }}_{\mathrm {w}}=\mu _{\mathrm {w}}^{\mathrm {e,0}}\,, \end{aligned}$$

8b $$\begin{aligned}&\mu _{\mathrm {i}}^{\mathrm {e}}={\bar{\mu }}_{\mathrm {i}}=\mu _{\mathrm {i}}^{\mathrm {e,0}}\,, \end{aligned}$$

where $\mu _{\mathrm {w}}^{\mathrm {e}}$ and $\mu _{\mathrm {i}}^{\mathrm {e}}$ are the EC chemical potentials of water and ion ${\mathrm {i}}$, coinciding with those of the bath ${\bar{\mu }}_{\mathrm {w}}$ and ${\bar{\mu }}_{\mathrm {i}}$, in turn supposed to be equal to the initial EC ones. Instead, in the presence of sealing TJs we impose the boundary conditions

9a $$\begin{aligned}&{\mathbf {J}}_{\mathrm {w}}^{\mathrm {e}}\cdot {\mathbf {N}}=0\,, \end{aligned}$$

9b $$\begin{aligned}&{\mathbf {J}}_{\mathrm {i}}^{\mathrm {e}}\cdot {\mathbf {N}}=0\,. \end{aligned}$$

We assign to Eq. (4a) the boundary condition

10 $$\begin{aligned} {\mathbf {D}}\cdot {\mathbf {N}}={\bar{D}}^{\mathrm {m}}\,, \end{aligned}$$

in which ${\bar{D}}^{\mathrm {m}}$ is the transmembrane electric displacement at the boundary, which is defined in Sect. 3.6.

As for Eq. (4b), in the absence of TJs we impose electrical equilibrium, that is,

11 $$\begin{aligned} \psi ^{\mathrm {e}}={\bar{\psi }}=0\,, \end{aligned}$$

where ${\bar{\psi }}$ is the electric potential of the bath, assumed to be zero. Instead, in the presence of TJs, we impose

12 $$\begin{aligned} {\mathbf {D}}^{\mathrm {e}}\cdot {\mathbf {N}}={\bar{D}}^{\mathrm {tj}}\,, \end{aligned}$$

with ${\bar{D}}^{\mathrm {tj}}$ denoting the electric displacement across the TJs, which is defined in Sect. 3.6.

While in the present work we focus on *endogenous* mechanobioelectricity, our framework may be also adopted to investigate the effect of an externally applied mechanical load or electric field, or of the exposure to an hypotonic or hypertonic environment, by enforcing appropriate boundary conditions.

### Thermodynamic restrictions

We follow the approach of Gurtin et al. (2010) for coupled problems of mechanics and species transport, suitably augmented to account for the electric charge of ions. Therefore, under isothermal conditions, the energy balance reads

13 $$\begin{aligned}&{\dot{U}}={\mathbf {P}}\cdot \dot{{\mathbf {F}}} +\varPhi _0\Bigl (\mu _{\mathrm {w}}{\dot{C}}_{\mathrm {w}}+\sum _{\mathrm {i}}\mu _{\mathrm {i}}{\dot{C}}_{\mathrm {i}}\Bigr ) +\varPhi _0^{\mathrm {e}}\Bigl (\mu _{\mathrm {w}}^{\mathrm {e}}{\dot{C}}_{\mathrm {w}}^{\mathrm {e}}+\sum _{\mathrm {i}}\mu _{\mathrm {i}}^{\mathrm {e}}{\dot{C}}_{\mathrm {i}}^{\mathrm {e}}\Bigr )\nonumber \\&\quad +{\mathbf {E}}\cdot \dot{{\mathbf {D}}} +{\mathbf {E}}^{\mathrm {e}}\cdot \dot{{\mathbf {D}}}^{\mathrm {e}} -{\mathbf {J}}_{\mathrm {w}}\cdot \nabla \mu _{\mathrm {w}} -\sum _{\mathrm {i}}{\mathbf {J}}_{\mathrm {i}}\cdot \nabla {\tilde{\mu }}_{\mathrm {i}} -{\mathbf {J}}_{\mathrm {w}}^{\mathrm {e}}\cdot \nabla \mu _{\mathrm {w}}^{\mathrm {e}}\nonumber \\&\quad -\sum _{\mathrm {i}}{\mathbf {J}}_{\mathrm {i}}^{\mathrm {e}}\cdot \nabla {\tilde{\mu }}_{\mathrm {i}}^{\mathrm {e}} -A^{\mathrm {c}}/V^{\mathrm {c}}\Bigl [J_{\mathrm {w}}^{\mathrm {m}}(\mu _{\mathrm {w}}^{\mathrm {e}}-\mu _{\mathrm {w}}) +\sum _{\mathrm {i}}J_{\mathrm {i}}^{\mathrm {m}}({\tilde{\mu }}_{\mathrm {i}}^{\mathrm {e}}-{\tilde{\mu }}_\mathrm {i})\Bigr ]\,, \end{aligned}$$

where *U* is the nominal internal energy density, ${\mathbf {F}}={\mathbf {I}}+\nabla {\mathbf {u}}$ is the deformation gradient (with ${\mathbf {I}}$ denoting the second-order identity tensor and $\nabla$ denoting the material gradient, such that $(\nabla {\mathbf {u}})_{iJ}=\partial u_i/\partial X_J$), $\mu _\mathrm {w}$ and $\mu _{\mathrm {i}}$ are the IC chemical potentials of water and ion ${\mathrm {i}}$,

14 $$\begin{aligned} {\mathbf {E}}=-\nabla \psi \,,\quad {\mathbf {E}}^{\mathrm {e}}=-\nabla \psi ^{\mathrm {e}} \end{aligned}$$

are the IC and EC nominal electric fields, and

15 $$\begin{aligned} {\tilde{\mu }}_{\mathrm {i}}=\mu _{\mathrm {i}}+Fz_{\mathrm {i}}\psi \,,\quad {\tilde{\mu }}_{\mathrm {i}}^{\mathrm {e}}=\mu _{\mathrm {i}}^{\mathrm {e}}+Fz_{\mathrm {i}}\psi ^{\mathrm {e}} \end{aligned}$$

are the IC and EC electrochemical potentials of ion ${\mathrm {i}}$.

Upon combining Eq. (13) with the second law of thermodynamics and introducing the nominal Helmholtz free energy density *W*, we obtain the free energy imbalance

16 $$\begin{aligned}&-{\dot{W}} +{\mathbf {P}}\cdot \dot{{\mathbf {F}}} +\varPhi _0\Bigl (\mu _{\mathrm {w}}{\dot{C}}_\mathrm {w}+\sum _{\mathrm {i}}\mu _{\mathrm {i}}{\dot{C}}_{\mathrm {i}}\Bigr )\nonumber \\&+\varPhi _0^{\mathrm {e}}\Bigl (\mu _{\mathrm {w}}^{\mathrm {e}}{\dot{C}}_{\mathrm {w}}^{\mathrm {e}}+\sum _{\mathrm {i}}\mu _{\mathrm {i}}^{\mathrm {e}}{\dot{C}}_{\mathrm {i}}^{\mathrm {e}}\Bigr ) +{\mathbf {E}}\cdot \dot{{\mathbf {D}}} +{\mathbf {E}}^{\mathrm {e}}\cdot \dot{{\mathbf {D}}}^{\mathrm {e}} -{\mathbf {J}}_{\mathrm {w}}\cdot \nabla \mu _{\mathrm {w}}\nonumber \\&-\sum _{\mathrm {i}}{\mathbf {J}}_{\mathrm {i}}\cdot \nabla {\tilde{\mu }}_{\mathrm {i}} -{\mathbf {J}}_{\mathrm {w}}^{\mathrm {e}}\cdot \nabla \mu _{\mathrm {w}}^{\mathrm {e}} -\sum _{\mathrm {i}}{\mathbf {J}}_{\mathrm {i}}^{\mathrm {e}}\cdot \nabla {\tilde{\mu }}_{\mathrm {i}}^{\mathrm {e}}\nonumber \\&\quad -A^{\mathrm {c}}/V^{\mathrm {c}}\Bigl [J_{\mathrm {w}}^{\mathrm {m}}(\mu _{\mathrm {w}}^{\mathrm {e}}-\mu _{\mathrm {w}}) +\sum _{\mathrm {i}}J_{\mathrm {i}}^{\mathrm {m}}({\tilde{\mu }}_{\mathrm {i}}^{\mathrm {e}}-{\tilde{\mu }}_{\mathrm {i}})\Bigr ] \ge 0\,. \end{aligned}$$

We assume that *W* is a function of the independent variables ${\mathbf {F}}$, $C_{\mathrm {w}}$, $C_{\mathrm {i}}$, $C_{\mathrm {w}}^{\mathrm {e}}$, $C_{\mathrm {i}}^{\mathrm {e}}$, ${\mathbf {D}}$, and ${\mathbf {D}}^{\mathrm {e}}$, such that Eq. (16) becomes

17 $$\begin{aligned}&\left( {\mathbf {P}}-\frac{\partial W}{\partial {\mathbf {F}}}\right) \cdot \dot{{\mathbf {F}}} +\left( \varPhi _0\mu _{\mathrm {w}}-\frac{\partial W}{\partial C_{\mathrm {w}}}\right) {\dot{C}}_{\mathrm {w}}\nonumber \\&+\sum _{\mathrm {i}}\left( \varPhi _0\mu _{\mathrm {i}}-\frac{\partial W}{\partial C_{\mathrm {i}}}\right) {\dot{C}}_{\mathrm {i}} +\left( \varPhi _0^{\mathrm {e}}\mu _{\mathrm {w}}^{\mathrm {e}}-\frac{\partial W}{\partial C_{\mathrm {w}}^{\mathrm {e}}}\right) {\dot{C}}_{\mathrm {w}}^{\mathrm {e}}\nonumber \\&+\sum _{\mathrm {i}}\left( \varPhi _0^{\mathrm {e}}\mu _{\mathrm {i}}^{\mathrm {e}}-\frac{\partial W}{\partial C_{\mathrm {i}}^{\mathrm {e}}}\right) {\dot{C}}_{\mathrm {i}}^{\mathrm {e}} +\left( {\mathbf {E}}-\frac{\partial W}{\partial {\mathbf {D}}}\right) \cdot \dot{{\mathbf {D}}}\nonumber \\&+\left( {\mathbf {E}}^{\mathrm {e}}-\frac{\partial W}{\partial {\mathbf {D}}^{\mathrm {e}}}\right) \cdot \dot{{\mathbf {D}}}^{\mathrm {e}} -{\mathbf {J}}_{\mathrm {w}}\cdot \nabla \mu _{\mathrm {w}}-\sum _{\mathrm {i}}{\mathbf {J}}_{\mathrm {i}}\cdot \nabla {\tilde{\mu }}_{\mathrm {i}}\nonumber \\&-{\mathbf {J}}_{\mathrm {w}}^{\mathrm {e}}\cdot \nabla \mu _{\mathrm {w}}^{\mathrm {e}}-\sum _{\mathrm {i}}{\mathbf {J}}_{\mathrm {i}}^{\mathrm {e}}\cdot \nabla {\tilde{\mu }}_{\mathrm {i}}^{\mathrm {e}}\nonumber \\&\quad -A^{\mathrm {c}}/V^{\mathrm {c}}\Bigl [J_{\mathrm {w}}^{\mathrm {m}}(\mu _{\mathrm {w}}^{\mathrm {e}}-\mu _{\mathrm {w}}) +\sum _{\mathrm {i}}J_{\mathrm {i}}^{\mathrm {m}}({\tilde{\mu }}_{\mathrm {i}}^{\mathrm {e}}-{\tilde{\mu }}_{\mathrm {i}})\Bigr ]\ge 0\,. \end{aligned}$$

By relying on the Coleman–Noll procedure, we obtain the following constitutive prescriptions:

18a $$\begin{aligned}&{\mathbf {P}}=\frac{\partial W}{\partial {\mathbf {F}}}\,, \end{aligned}$$

18b $$\begin{aligned}&\mu _{\mathrm {w}}=\frac{1}{\varPhi _0}\frac{\partial W}{\partial C_{\mathrm {w}}}\,,\quad \mu _{\mathrm {w}}^{\mathrm {e}}=\frac{1}{\varPhi _0^{\mathrm {e}}}\frac{\partial W}{\partial C_{\mathrm {w}}^{\mathrm {e}}}\,, \end{aligned}$$

18c $$\begin{aligned}&\mu _{\mathrm {i}}=\frac{1}{\varPhi _0}\frac{\partial W}{\partial C_{\mathrm {i}}}\,,\quad \mu _{\mathrm {i}}^{\mathrm {e}}=\frac{1}{\varPhi _0^{\mathrm {e}}}\frac{\partial W}{\partial C_{\mathrm {i}}^{\mathrm {e}}}\,, \end{aligned}$$

18d $$\begin{aligned}&{\mathbf {E}}=\frac{\partial W}{\partial {\mathbf {D}}}\,,\quad {\mathbf {E}}^{\mathrm {e}}=\frac{\partial W}{\partial {\mathbf {D}}^{\mathrm {e}}}\,. \end{aligned}$$

Consequently, Eq. (17) reduces to the dissipation inequality

19 $$\begin{aligned}&-{\mathbf {J}}_{\mathrm {w}}\cdot \nabla \mu _{\mathrm {w}}-\sum _{\mathrm {i}}{\mathbf {J}}_{\mathrm {i}}\cdot \nabla {\tilde{\mu }}_{\mathrm {i}} -{\mathbf {J}}_{\mathrm {w}}^{\mathrm {e}}\cdot \nabla \mu _{\mathrm {w}}^{\mathrm {e}}-\sum _{\mathrm {i}}{\mathbf {J}}_{\mathrm {i}}^{\mathrm {e}}\cdot \nabla {\tilde{\mu }}_{\mathrm {i}}^{\mathrm {e}}\nonumber \\&\quad -A^{\mathrm {c}}/V^{\mathrm {c}}\Bigl [J_{\mathrm {w}}^{\mathrm {m}}(\mu _{\mathrm {w}}^{\mathrm {e}}-\mu _{\mathrm {w}}) +\sum _{\mathrm {i}}J_{\mathrm {i}}^{\mathrm {m}}({\tilde{\mu }}_{\mathrm {i}}^{\mathrm {e}}-{\tilde{\mu }}_{\mathrm {i}})\Bigr ]\ge 0\,. \end{aligned}$$

We remark that the terms in the second line are local dissipation terms, arising from the exchange of water and ions across the cell membrane within the same material point.

Since water and ions share the same IC and EC spaces, we assume that each IC or EC flux is affected by the chemical potential gradient of water and by the electrochemical potential gradients of all mobile ions, that is,

20a $$\begin{aligned}&{\mathbf {J}}_{\mathrm {w}}=-{\mathbf {M}}_{\mathrm {ww}}\nabla \mu _{\mathrm {w}}-\sum _{\mathrm {i}}{\mathbf {M}}_{\mathrm {wi}}\nabla {\tilde{\mu }}_{\mathrm {i}}\,, \end{aligned}$$

20b $$\begin{aligned}&{\mathbf {J}}_{\mathrm {i}}=-{\mathbf {M}}_{\mathrm {wi}}\nabla \mu _{\mathrm {w}}-{\mathbf {M}}_{\mathrm {ii}}\nabla {\tilde{\mu }}_{\mathrm {i}}-\sum _{\mathrm {j}}{\mathbf {M}}_{\mathrm {ij}}\nabla {\tilde{\mu }}_{\mathrm {j}}\,,\quad {\mathrm {j}}\ne {\mathrm {i}}\,, \end{aligned}$$

20c $$\begin{aligned}&{\mathbf {J}}_{\mathrm {w}}^{\mathrm {e}}=-{\mathbf {M}}_{\mathrm {ww}}^{\mathrm {e}}\nabla \mu _{\mathrm {w}}^{\mathrm {e}}-\sum _{\mathrm {i}}{\mathbf {M}}_{\mathrm {wi}}^{\mathrm {e}}\nabla {\tilde{\mu }}_{\mathrm {i}}^{\mathrm {e}}\,, \end{aligned}$$

20d $$\begin{aligned}&{\mathbf {J}}_{\mathrm {i}}^{\mathrm {e}}=-{\mathbf {M}}_{\mathrm {wi}}^{\mathrm {e}}\nabla \mu _{\mathrm {w}}^{\mathrm {e}}-{\mathbf {M}}_{\mathrm {ii}}^{\mathrm {e}}\nabla {\tilde{\mu }}_{\mathrm {i}}^{\mathrm {e}}-\sum _{\mathrm {j}}{\mathbf {M}}_{\mathrm {ij}}^{\mathrm {e}}\nabla {\tilde{\mu }}_{\mathrm {j}}^{\mathrm {e}}\,,\quad {\mathrm {j}}\ne {\mathrm {i}}\,, \end{aligned}$$

where the constitutive operators can be collected into the symmetric (Onsager 1931) mobility matrices

21a $$\begin{aligned}&\varvec{{\mathcal {M}}}=\begin{bmatrix} {\mathbf {M}}_{\mathrm {ww}} &{} {\mathbf {M}}_{\mathrm {w1}} &{} {\mathbf {M}}_{\mathrm {w2}} &{} \dots \\ {\mathbf {M}}_{\mathrm {w1}} &{} {\mathbf {M}}_{\mathrm {11}} &{} {\mathbf {M}}_{\mathrm {12}} &{} \dots \\ {\mathbf {M}}_{\mathrm {w2}} &{} {\mathbf {M}}_{\mathrm {12}} &{} {\mathbf {M}}_{\mathrm {22}} &{} \dots \\ \vdots &{} \vdots &{} \vdots &{} \ddots \end{bmatrix}\,, \end{aligned}$$

21b $$\begin{aligned}&\varvec{{\mathcal {M}}}^{\mathrm {e}}=\begin{bmatrix} {\mathbf {M}}_{\mathrm {ww}}^{\mathrm {e}} &{} {\mathbf {M}}_{\mathrm {w1}}^{\mathrm {e}} &{} {\mathbf {M}}_{\mathrm {w2}}^{\mathrm {e}} &{} \dots \\ {\mathbf {M}}_{\mathrm {w1}}^{\mathrm {e}} &{} {\mathbf {M}}_{\mathrm {11}}^{\mathrm {e}} &{} {\mathbf {M}}_{\mathrm {12}}^{\mathrm {e}} &{} \dots \\ {\mathbf {M}}_{\mathrm {w2}}^{\mathrm {e}} &{} {\mathbf {M}}_{\mathrm {12}}^{\mathrm {e}} &{} {\mathbf {M}}_{\mathrm {22}}^{\mathrm {e}} &{} \dots \\ \vdots &{} \vdots &{} \vdots &{} \ddots \end{bmatrix}\,, \end{aligned}$$

in which the off-diagonal entries account for so-called *cross-diffusion* (Vanag and Epstein 2009).

Conversely, aquaporins and ion channels are specific for water and ion transport. Therefore, the transmembrane fluxes of water and ion ${\mathrm {i}}$ only depend on the difference between the EC and IC chemical potentials of water and electrochemical potentials of ion ${\mathrm {i}}$, respectively:

22a $$\begin{aligned}&J_{\mathrm {w}}^{\mathrm {m}}=-M_{\mathrm {w}}^{\mathrm {m}}(\mu _{\mathrm {w}}^{\mathrm {e}}-\mu _{\mathrm {w}})\,, \end{aligned}$$

22b $$\begin{aligned}&J_{\mathrm {i}}^{\mathrm {m}}=-M_{\mathrm {i}}^{\mathrm {m}}({\tilde{\mu }}_{\mathrm {i}}^{\mathrm {e}}-{\tilde{\mu }}_{\mathrm {i}})\,. \end{aligned}$$

We assume the mobility matrices $\varvec{{\mathcal {M}}}$ and $\varvec{{\mathcal {M}}}^{\mathrm {e}}$ to be positive definite and the mobility coefficients $M_{\mathrm {w}}^{\mathrm {m}}$ and $M_{\mathrm {i}}^{\mathrm {m}}$ to be positive, in order to fulfill Eq. (19), as detailed in Sect. 3.7.

### Free energy density

We choose the following additive decomposition for the free energy density:

23 $$\begin{aligned} W=W_{\mathrm {mec}}({\mathbf {F}})&+W_{\mathrm {mix}}(C_{\mathrm {w}},C_{\mathrm {i}})+W_{\mathrm {mix}}^{\mathrm {e}}(C_{\mathrm {w}}^{\mathrm {e}},C_{\mathrm {i}}^{\mathrm {e}})\nonumber \\&\quad +W_{\mathrm {pol}}({\mathbf {F}},{\mathbf {D}})+W_{\mathrm {pol}}^{\mathrm {e}}({\mathbf {F}},{\mathbf {D}}^{\mathrm {e}})\,, \end{aligned}$$

where $W_{\mathrm {mec}}$ accounts for the elasticity of the solid network, $W_{\mathrm {mix}}$ and $W_{\mathrm {mix}}^{\mathrm {e}}$ account for the mixing of water and ions in the IC and EC spaces, and $W_{\mathrm {pol}}$ and $W_{\mathrm {pol}}^{\mathrm {e}}$ account for the dielectric polarization of the IC and EC spaces.

We adopt for $W_{\mathrm {mec}}$ the compressible Neo-Hookean model proposed by Simo and Pister (1984):

24 $$\begin{aligned} W_{\mathrm {mec}}({\mathbf {F}})=\frac{G}{2}({\mathrm {tr}}\,{\mathbf {C}}-3)-G\ln J+\frac{1}{2}\lambda \ln ^2 J\,, \end{aligned}$$

where $\lambda$ and *G* are the first and second Lamé parameters of the solid network, ${\mathbf {C}}={\mathbf {F}}^{\mathrm {T}}{\mathbf {F}}$ is the right Cauchy–Green deformation tensor, and

25 $$\begin{aligned} J={\mathrm {det}}\,{\mathbf {F}} \end{aligned}$$

is the Jacobian, that is, the volume ratio.

We assume the IC and EC solutions of water and ions to be ideal, such that $W_{\mathrm {mix}}$ and $W_{\mathrm {mix}}^{\mathrm {e}}$ are purely entropic and read (Ateshian 2007)

26a $$\begin{aligned}&W_{\mathrm {mix}}(C_{\mathrm {w}},C_{\mathrm {i}})={\mathcal {R}}T\varPhi _0\nonumber \\&\quad \times \left( C_{\mathrm {w}}\ln \frac{C_{\mathrm {w}}}{C_{\mathrm {w}}+\sum _{\mathrm {j}}C_{\mathrm {j}}}+\sum _{\mathrm {i}}C_{\mathrm {i}}\ln \frac{C_{\mathrm {i}}}{C_{\mathrm {w}}+\sum _{\mathrm {j}}C_{\mathrm {j}}}\right) \,, \end{aligned}$$

26b $$\begin{aligned}&W_{\mathrm {mix}}^{\mathrm {e}}(C_{\mathrm {w}}^{\mathrm {e}},C_{\mathrm {i}}^{\mathrm {e}})={\mathcal {R}}T\varPhi _0^{\mathrm {e}}\nonumber \\&\quad \times \left( C_{\mathrm {w}}^{\mathrm {e}}\ln \frac{C_{\mathrm {w}}^{\mathrm {e}}}{C_{\mathrm {w}}^{\mathrm {e}}+\sum _{\mathrm {j}}C_{\mathrm {j}}^{\mathrm {e}}}+\sum _{\mathrm {i}}C_{\mathrm {i}}^{\mathrm {e}}\ln \frac{C_{\mathrm {i}}^{\mathrm {e}}}{C_{\mathrm {w}}^{\mathrm {e}}+\sum _{\mathrm {j}}C_{\mathrm {j}}^{\mathrm {e}}}\right) \,, \end{aligned}$$

where ${\mathcal {R}}$ is the gas constant and *T* is the absolute temperature. We note that $W_{\mathrm {mix}}$ and $W_{\mathrm {mix}}^{\mathrm {e}}$ account for both mobile and fixed ions, also the latter being part of the IC and EC solutions. We further hypothesize that the IC and EC solutions are dilute, that is, $\sum _{\mathrm {i}}C_{\mathrm {i}}\ll C_{\mathrm {w}}$ and $\sum _{\mathrm {i}}C_{\mathrm {i}}^{\mathrm {e}}\ll C_{\mathrm {w}}^{\mathrm {e}}$, such that we may rewrite Eqs. (26) as

27a $$\begin{aligned}&W_{\mathrm {mix}}(C_{\mathrm {w}},C_{\mathrm {i}})={\mathcal {R}}T\varPhi _0\sum _{\mathrm {i}}C_{\mathrm {i}}\left( \ln \frac{C_{\mathrm {i}}}{C_{\mathrm {w}}}-1\right) \,, \end{aligned}$$

27b $$\begin{aligned}&W_{\mathrm {mix}}^{\mathrm {e}}(C_{\mathrm {w}}^{\mathrm {e}},C_{\mathrm {i}}^{\mathrm {e}})={\mathcal {R}}T\varPhi _0^{\mathrm {e}}\sum _{\mathrm {i}}C_{\mathrm {i}}^{\mathrm {e}}\left( \ln \frac{C_{\mathrm {i}}^{\mathrm {e}}}{C_{\mathrm {w}}^{\mathrm {e}}}-1\right) \,. \end{aligned}$$

Finally, we treat the IC and EC solutions as ideal dielectrics, such that $W_{\mathrm {pol}}$ and $W_{\mathrm {pol}}^{\mathrm {e}}$ read (Hong et al. 2010)

28a $$\begin{aligned}&W_{\mathrm {pol}}({\mathbf {F}},{\mathbf {D}})=\frac{|{\mathbf {F}}{\mathbf {D}}|^2}{2\varepsilon _0\varepsilon _{\mathrm {r}} J}\,, \end{aligned}$$

28b $$\begin{aligned}&W_{\mathrm {pol}}^{\mathrm {e}}({\mathbf {F}},{\mathbf {D}}^{\mathrm {e}})=\frac{|{\mathbf {F}}{\mathbf {D}}^{\mathrm {e}}|^2}{2\varepsilon _0\varepsilon _{\mathrm {r}} J}\,, \end{aligned}$$

in which $\varepsilon _0$ is the vacuum permittivity and $\varepsilon _{\mathrm {r}}$ is the relative permittivity of the IC and EC solutions, assumed to coincide with that of water given their diluteness.

### Constraint on the volume ratio

We assume that the solid network, water, and ions are incompressible, such that the volume ratio of Eq. (25) is inextricably related to the redistribution of water and ions, namely

29 $$\begin{aligned} J&=1+\varPhi _0\Bigl (v_{\mathrm {w}}C_{\mathrm {w}}+\sum _{\mathrm {i}}v_{\mathrm {i}}C_{\mathrm {i}}-1\Bigr )\nonumber \\&\quad +\varPhi _0^{\mathrm {e}}\Bigl (v_{\mathrm {w}}C_{\mathrm {w}}^{\mathrm {e}}+\sum _{\mathrm {i}}v_{\mathrm {i}}C_{\mathrm {i}}^{\mathrm {e}}-1\Bigr )\,, \end{aligned}$$

where $v_{\mathrm {w}}$ and $v_{\mathrm {i}}$ are the molar volumes of water and ion ${\mathrm {i}}$. In the limit of dilute IC and EC solutions, Eq. (29) reduces to

30 $$\begin{aligned} J=1+\varPhi _0\Bigl (v_{\mathrm {w}}C_{\mathrm {w}}-1\Bigr )+\varPhi _0^{\mathrm {e}}\Bigl (v_{\mathrm {w}}C_{\mathrm {w}}^{\mathrm {e}}-1\Bigr )\,, \end{aligned}$$

implying that

31 $$\begin{aligned} C_{\mathrm {w}}^0=C_{\mathrm {w}}^{\mathrm {e,0}}=\frac{1}{v_{\mathrm {w}}}\,, \end{aligned}$$

to be replaced in Eq. (6a). Moreover,

32 $$\begin{aligned} c_{\mathrm {i}}=\frac{C_{\mathrm {i}}}{v_{\mathrm {w}}C_{\mathrm {w}}}\,,\quad c_{\mathrm {i}}^{\mathrm {e}}=\frac{C_{\mathrm {i}}^{\mathrm {e}}}{v_{\mathrm {w}}C_{\mathrm {w}}^{\mathrm {e}}} \end{aligned}$$

are the IC and EC molar concentrations of ion ${\mathrm {i}}$ per unit *current* volume of the IC and EC spaces.

In order to impose the constraint (30), we modify the free energy density (23) as (Hong et al. 2010)

33 $$\begin{aligned} W&=W_{\mathrm {mec}}({\mathbf {F}})+W_{\mathrm {mix}}(C_{\mathrm {w}},C_{\mathrm {i}})+W_\mathrm {mix}^{\mathrm {e}}(C_{\mathrm {w}}^{\mathrm {e}},C_{\mathrm {i}}^{\mathrm {e}})\nonumber \\&\quad +W_{\mathrm {pol}}({\mathbf {F}},{\mathbf {D}})+W_{\mathrm {pol}}^{\mathrm {e}}({\mathbf {F}},{\mathbf {D}}^{\mathrm {e}})\nonumber \\&\quad +p_{\mathrm {w}}\left[ 1+\varPhi _0\Bigl (v_{\mathrm {w}}C_{\mathrm {w}}-1\Bigr )+\varPhi _0^{\mathrm {e}}\Bigl (v_{\mathrm {w}}C_{\mathrm {w}}^{\mathrm {e}}-1\Bigr )-J\right] \,, \end{aligned}$$

where $p_{\mathrm {w}}$ is a Lagrange multiplier field assuming the role of water pressure. For later developments, we rearrange Eq. (30) for the IC water concentration:

34 $$\begin{aligned} C_{\mathrm {w}}=\frac{1}{v_{\mathrm {w}}}+\frac{1}{\varPhi _0v_{\mathrm {w}}}\left[ J-1-\varPhi _0^{\mathrm {e}}\Bigl (v_{\mathrm {w}}C_{\mathrm {w}}^{\mathrm {e}}-1\Bigr )\right] \,. \end{aligned}$$

Notably, this operation removes $C_{\mathrm {w}}$ from the list of the independent variables, in favor of the independent variable $p_{\mathrm {w}}$ introduced by Eq. (33).

### Conservative constitutive laws

We obtain the nominal stress ${\mathbf {P}}$ by combining Eqs. (18a), (24), and (33):

35 $$\begin{aligned} {\mathbf {P}}&=\underbrace{G({\mathbf {F}}-{\mathbf {F}}^{\mathrm {-T}})+\lambda \ln \,J{\mathbf {F}}^\mathrm {-T}}_{\displaystyle {{\mathbf {P}}_{\mathrm {mec}}}} \underbrace{-p_{\mathrm {w}}J{\mathbf {F}}^{\mathrm {-T}}}_{\displaystyle {{\mathbf {P}}_{\mathrm {w}}}}\nonumber \\&\quad +\underbrace{\frac{1}{2\varepsilon _0\varepsilon _{\mathrm {r}} J}\Bigl [2{\mathbf {F}}({\mathbf {D}}\otimes {\mathbf {D}})-{\mathbf {C}}\cdot ({\mathbf {D}}\otimes {\mathbf {D}}){\mathbf {F}}^{\mathrm {-T}}\Bigr ]}_{\displaystyle {{\mathbf {P}}_{\mathrm {pol}}}}\nonumber \\&\quad +\underbrace{\frac{1}{2\varepsilon _0\varepsilon _{\mathrm {r}} J}\Bigl [2{\mathbf {F}}({\mathbf {D}}^{\mathrm {e}}\otimes {\mathbf {D}}^{\mathrm {e}})-{\mathbf {C}}\cdot ({\mathbf {D}}^{\mathrm {e}}\otimes {\mathbf {D}}^{\mathrm {e}}){\mathbf {F}}^{\mathrm {-T}}\Bigr ]}_{\displaystyle {{\mathbf {P}}_{\mathrm {pol}}^{\mathrm {e}}}}\,, \end{aligned}$$

where $\otimes$ denotes the tensor product, such that $({\mathbf {D}}\otimes {\mathbf {D}})_{IJ}=D_ID_J$. The stresses ${\mathbf {P}}_{\mathrm {w}}$, ${\mathbf {P}}_{\mathrm {pol}}$, and ${\mathbf {P}}_{\mathrm {pol}}^{\mathrm {e}}$ could be regarded as active stresses (or *eigenstresses*), to be balanced by ${\mathbf {P}}_{\mathrm {mec}}$ through equilibrium (1). The corresponding Cauchy stress is

36 $$\begin{aligned} \varvec{\sigma }&=\frac{1}{J}{\mathbf {P}}{\mathbf {F}}^\mathrm {T}=\underbrace{\frac{1}{J}\Bigl [G({\mathbf {b}}-{\mathbf {I}})+\lambda \ln J{\mathbf {I}}\Bigr ]}_{\displaystyle {\varvec{\sigma }_{\mathrm {mec}}}} \underbrace{-p_{\mathrm {w}}{\mathbf {I}}}_{\displaystyle {\varvec{\sigma }_{\mathrm {w}}}}\nonumber \\&\quad +\underbrace{\frac{1}{2\varepsilon _0\varepsilon _{\mathrm {r}}}\Bigl [2{\mathbf {d}}\otimes {\mathbf {d}}-({\mathbf {d}}\cdot {\mathbf {d}}){\mathbf {I}}\Bigr ]}_{\displaystyle {\varvec{\sigma }_{\mathrm {pol}}}}\nonumber \\&\quad +\underbrace{\frac{1}{2\varepsilon _0\varepsilon _{\mathrm {r}}}\Bigl [2{\mathbf {d}}^{\mathrm {e}}\otimes {\mathbf {d}}^{\mathrm {e}}-({\mathbf {d}}^{\mathrm {e}}\cdot {\mathbf {d}}^{\mathrm {e}}){\mathbf {I}}\Bigr ]}_{\displaystyle {\varvec{\sigma }_{\mathrm {pol}}^{\mathrm {e}}}}\,, \end{aligned}$$

where ${\mathbf {b}}={\mathbf {F}}{\mathbf {F}}^{\mathrm {T}}$ is the left Cauchy–Green deformation tensor and ${\mathbf {d}}=J^{-1}{\mathbf {F}}{\mathbf {D}}$ and ${\mathbf {d}}^{\mathrm {e}}=J^{-1}{\mathbf {F}}{\mathbf {D}}^{\mathrm {e}}$ are the IC and EC current electric displacements. We define the pressure as

37 $$\begin{aligned} p&=-\frac{1}{3}{\mathrm {tr}}\,\varvec{\sigma } =\underbrace{-\frac{1}{J}\left[ G\left( \frac{1}{3}{\mathrm {tr}}\,{\mathbf {b}}-1\right) +\lambda \ln J\right] }_{\displaystyle {p_{\mathrm {mec}}}}+p_{\mathrm {w}}\nonumber \\&\quad +\underbrace{\frac{1}{6\varepsilon _0\varepsilon _{\mathrm {r}}}|{\mathbf {d}}|^2}_{\displaystyle {p_{\mathrm {pol}}}} +\underbrace{\frac{1}{6\varepsilon _0\varepsilon _{\mathrm {r}}}|{\mathbf {d}}^{\mathrm {e}}|^2}_{\displaystyle {p_{\mathrm {pol}}^{\mathrm {e}}}}\,, \end{aligned}$$

adopting the convention that each contribution to *p* is positive if compressive.

By using Eqs. (18b), (27), and (33), we obtain the following IC and EC chemical potentials of water:

38 $$\begin{aligned} \mu _{\mathrm {w}}=-{\mathcal {R}}T\frac{C}{C_{\mathrm {w}}}+v_{\mathrm {w}}p_{\mathrm {w}}\,,\quad \mu _{\mathrm {w}}^{\mathrm {e}}=-{\mathcal {R}}T\frac{C^{\mathrm {e}}}{C_{\mathrm {w}}^{\mathrm {e}}}+v_{\mathrm {w}}p_{\mathrm {w}}\,, \end{aligned}$$

where

39 $$\begin{aligned} C=\sum _{\mathrm {i}}C_{\mathrm {i}}\,,\quad C^{\mathrm {e}}=\sum _{\mathrm {i}}C_{\mathrm {i}}^{\mathrm {e}} \end{aligned}$$

are the IC and EC osmotic concentrations. We remark that $\mu _{\mathrm {w}}$ and $\mu _{\mathrm {w}}^{\mathrm {e}}$ are affected by both the IC and EC osmotic pressures ${\mathcal {R}}TC$ and ${\mathcal {R}}TC^{\mathrm {e}}$ and the water pressure $p_{\mathrm {w}}$. Boundary condition (8a) may now be explicited for the independent variable $C_{\mathrm {w}}^{\mathrm {e}}$, thus reading

40 $$\begin{aligned} C_{\mathrm {w}}^{\mathrm {e}}=\frac{{\mathcal {R}}TC^{\mathrm {e}}}{v_{\mathrm {w}}\left( {\mathcal {R}}TC^{\mathrm {e,0}}+p_{\mathrm {w}}\right) }\,, \end{aligned}$$

where we have used

41 $$\begin{aligned} p_{\mathrm {w}}^0=0\,. \end{aligned}$$

By combining Eqs. (15), (18c), (27), and (33) we obtain the following IC and EC electrochemical potentials of ion ${\mathrm {i}}$:

42 $$\begin{aligned} {\tilde{\mu }}_{\mathrm {i}}={\mathcal {R}}T\,{\mathrm {ln}}\frac{C_{\mathrm {i}}}{C_{\mathrm {w}}}+Fz_\mathrm {i}\psi \,,\quad {\tilde{\mu }}_{\mathrm {i}}^{\mathrm {e}}={\mathcal {R}}T\,{\mathrm {ln}}\frac{C_{\mathrm {i}}^{\mathrm {e}}}{C_{\mathrm {w}}^{\mathrm {e}}}+Fz_{\mathrm {i}}\psi ^{\mathrm {e}}\,. \end{aligned}$$

Boundary condition (8b) may now be explicited for the independent variable $C_{\mathrm {i}}^{\mathrm {e}}$, thus reading

43 $$\begin{aligned} C_{\mathrm {i}}^{\mathrm {e}}=v_{\mathrm {w}}C_{\mathrm {i}}^{\mathrm {e,0}}C_{\mathrm {w}}^{\mathrm {e}}\,. \end{aligned}$$

Finally, the usage of Eqs. (14), (18d), (28), and (33) provides the IC and EC nominal electric fields, whose inversion results in the IC and EC nominal electric displacements

44 $$\begin{aligned} {\mathbf {D}}=-\varepsilon _0\varepsilon _{\mathrm {r}} J{\mathbf {C}}^{-1}\nabla \psi \,,\quad {\mathbf {D}}^{\mathrm {e}}=-\varepsilon _0\varepsilon _{\mathrm {r}} J{\mathbf {C}}^{-1}\nabla \psi ^{\mathrm {e}}\,. \end{aligned}$$

Similarly, the electric displacements at the boundary across the cell membranes and the TJs in Eqs. (10) and (12) are given by

45a $$\begin{aligned}&{\bar{D}}^{\mathrm {m}}=\varepsilon _0\varepsilon _{\mathrm {r}}^{\mathrm {m}}\frac{\psi -{\bar{\psi }}}{T^{\mathrm {m}}}=\varepsilon _0\varepsilon _{\mathrm {r}}^{\mathrm {m}}\frac{\psi }{T^{\mathrm {m}}}\,, \end{aligned}$$

45b $$\begin{aligned}&{\bar{D}}^{\mathrm {tj}}=\varepsilon _0\varepsilon _{\mathrm {r}}^{\mathrm {tj}}\frac{\psi ^{\mathrm {e}}-{\bar{\psi }}}{T^{\mathrm {tj}}}=\varepsilon _0\varepsilon _{\mathrm {r}}^{\mathrm {tj}}\frac{\psi ^{\mathrm {e}}}{T^{\mathrm {tj}}}\,, \end{aligned}$$

in which $\varepsilon _{\mathrm {r}}^{\mathrm {m}}$ and $\varepsilon _{\mathrm {r}}^{\mathrm {tj}}$ and $T^{\mathrm {m}}$ and $T^{\mathrm {tj}}$ are the membrane and TJ relative permittivities and thicknesses, respectively. Eqs. (45) neglect the local deformation of cell membranes and TJs.

### Dissipative constitutive laws

We choose the following form for the IC and EC mobility matrices of Eqs. (21):

46a $$\begin{aligned} \varvec{{\mathcal {M}}}&=\frac{1}{{\mathcal {R}}T}{\mathbf {C}}^{-1}\nonumber \\&\quad \times \small {\begin{bmatrix} D_{\mathrm {w}}C_{\mathrm {w}} &{} D_{\mathrm {w}}C_1 &{} D_{\mathrm {w}}C_2 &{} \dots \\ \\ D_{\mathrm {w}}C_1 &{} \left( D_{\mathrm {w}}\dfrac{C_1}{C_{\mathrm {w}}}+D_1\right) C_1 &{} D_{\mathrm {w}}\dfrac{C_1C_2}{C_{\mathrm {w}}} &{} \dots \\ \\ D_{\mathrm {w}}C_2 &{} D_{\mathrm {w}}\dfrac{C_1C_2}{C_{\mathrm {w}}} &{} \left( D_{\mathrm {w}}\dfrac{C_2}{C_{\mathrm {w}}}+D_2\right) C_2 &{} \dots \\ \\ \vdots &{} \vdots &{} \vdots &{} \ddots \end{bmatrix}}\,, \end{aligned}$$

46b $$\begin{aligned} \varvec{{\mathcal {M}}}^{\mathrm {e}}&=\frac{1}{{\mathcal {R}}T}{\mathbf {C}}^{-1}\nonumber \\&\quad \times \small {\begin{bmatrix} D_{\mathrm {w}}^{\mathrm {e}}C_{\mathrm {w}}^{\mathrm {e}} &{} D_{\mathrm {w}}^{\mathrm {e}}C_1^{\mathrm {e}} &{} D_{\mathrm {w}}^{\mathrm {e}}C_2^{\mathrm {e}} &{} \dots \\ \\ D_{\mathrm {w}}^{\mathrm {e}}C_1^{\mathrm {e}} &{} \left( D_{\mathrm {w}}^{\mathrm {e}}\dfrac{C_1^{\mathrm {e}}}{C_{\mathrm {w}}^{\mathrm {e}}}+D_1^{\mathrm {e}}\right) C_1^{\mathrm {e}} &{} D_{\mathrm {w}}^{\mathrm {e}}\dfrac{C_1^{\mathrm {e}}C_2^{\mathrm {e}}}{C_{\mathrm {w}}^{\mathrm {e}}} &{} \dots \\ \\ D_{\mathrm {w}}^{\mathrm {e}}C_2^{\mathrm {e}} &{} D_{\mathrm {w}}^{\mathrm {e}}\dfrac{C_1^{\mathrm {e}}C_2^{\mathrm {e}}}{C_{\mathrm {w}}^{\mathrm {e}}} &{} \left( D_{\mathrm {w}}^{\mathrm {e}}\dfrac{C_2^{\mathrm {e}}}{C_{\mathrm {w}}^{\mathrm {e}}}+D_2^{\mathrm {e}}\right) C_2^{\mathrm {e}} &{} \dots \\ \\ \vdots &{} \vdots &{} \vdots &{} \ddots \end{bmatrix}}\,, \end{aligned}$$

in which $D_{\mathrm {w}}$ and $D_{\mathrm {w}}^{\mathrm {e}}$ are the water diffusivities in the IC and EC spaces, while $D_{\mathrm {i}}$ and $D_{\mathrm {i}}^{\mathrm {e}}$ are the diffusivities of ion ${\mathrm {i}}$ in the IC and EC water. As for the transmembrane mobility coefficients of Eqs. (22), by still neglecting the local deformation of the membrane, we adopt the forms

47a $$\begin{aligned}&M_{\mathrm {w}}^{\mathrm {m}}=\frac{D_{\mathrm {w}}^{\mathrm {m}}}{{\mathcal {R}}T}\frac{C_{\mathrm {w}}+C_{\mathrm {w}}^{\mathrm {e}}}{2T^{\mathrm {m}}}\,, \end{aligned}$$

47b $$\begin{aligned}&M_{\mathrm {i}}^{\mathrm {m}}=\frac{D_{\mathrm {i}}^{\mathrm {m}}}{{\mathcal {R}}T}\frac{C_{\mathrm {i}}+C_{\mathrm {i}}^{\mathrm {e}}}{2T^{\mathrm {m}}}\,, \end{aligned}$$

where $D_{\mathrm {w}}^{\mathrm {m}}$ and $D_{\mathrm {i}}^{\mathrm {m}}$ are the transmembrane diffusivities of water and ion ${\mathrm {i}}$. The mobility matrices $\varvec{{\mathcal {M}}}$ and $\varvec{{\mathcal {M}}}^{\mathrm {e}}$ are positive definite and the mobility coefficients $M_{\mathrm {w}}^{\mathrm {m}}$ and $M_{\mathrm {i}}^{\mathrm {m}}$ are positive for nonzero diffusivities and concentrations, thus ensuring the validity of Eq. (19).

Substituting Eqs. (46) into Eqs. (20) leads to

48a $$\begin{aligned}&{\mathbf {J}}_{\mathrm {w}}=-\frac{D_{\mathrm {w}}}{{\mathcal {R}}T}{\mathbf {C}}^{-1}\left[ C_{\mathrm {w}}\nabla \mu _{\mathrm {w}}+\sum _{\mathrm {i}}C_{\mathrm {i}}\nabla {\tilde{\mu }}_{\mathrm {i}}\right] \,, \end{aligned}$$

48b $$\begin{aligned}&{\mathbf {J}}_{\mathrm {i}}=\frac{C_{\mathrm {i}}}{C_{\mathrm {w}}}{\mathbf {J}}_{\mathrm {w}}-\frac{D_{\mathrm {i}}}{{\mathcal {R}}T}{\mathbf {C}}^{-1}C_{\mathrm {i}}\nabla {\tilde{\mu }}_{\mathrm {i}}\,, \end{aligned}$$

48c $$\begin{aligned}&{\mathbf {J}}_{\mathrm {w}}^{\mathrm {e}}=-\frac{D_{\mathrm {w}}^{\mathrm {e}}}{{\mathcal {R}}T}{\mathbf {C}}^{-1}\left[ C_{\mathrm {w}}^{\mathrm {e}}\nabla \mu _{\mathrm {w}}^{\mathrm {e}}+\sum _{\mathrm {i}}C_{\mathrm {i}}^{\mathrm {e}}\nabla {\tilde{\mu }}_{\mathrm {i}}^{\mathrm {e}}\right] \,, \end{aligned}$$

48d $$\begin{aligned}&{\mathbf {J}}_{\mathrm {i}}^{\mathrm {e}}=\frac{C_{\mathrm {i}}^{\mathrm {e}}}{C_{\mathrm {w}}^{\mathrm {e}}}{\mathbf {J}}_{\mathrm {w}}^{\mathrm {e}}-\frac{D_{\mathrm {i}}^{\mathrm {e}}}{{\mathcal {R}}T}{\mathbf {C}}^{-1}C_{\mathrm {i}}^{\mathrm {e}}\nabla {\tilde{\mu }}_{\mathrm {i}}^{\mathrm {e}}\,. \end{aligned}$$

In the perspective of mixture theory, one would obtain the same expressions by relying on the individual momentum balances for water and ions in the IC and EC spaces separately, and assuming that the friction between the different ion species and between the ions and the solid network in either the IC or EC space is negligible, given the diluteness of the solutions (Huyghe and Janssen 1997).

Combining Eqs. (38), (42), and (48) provides the following IC and EC fluxes:

49a $$\begin{aligned} {\mathbf {J}}_{\mathrm {w}}=-D_{\mathrm {w}}{\mathbf {C}}^{-1}\Biggl [\frac{v_{\mathrm {w}}C_{\mathrm {w}}}{{\mathcal {R}}T}\nabla p_{\mathrm {w}}+\frac{F}{{\mathcal {R}}T}\sum _{\mathrm {i}}z_{\mathrm {i}}C_{\mathrm {i}}\nabla \psi \nonumber \\\quad +\frac{\sum _{{\mathrm {j}}\ne {\mathrm{i}}}C_{\mathrm {j}}}{C_{\mathrm {w}}}\nabla C_{\mathrm {w}}\Biggr ]\,, \end{aligned}$$

49b $$\begin{aligned} {\mathbf {J}}_{\mathrm {i}}&=\frac{C_{\mathrm {i}}}{C_{\mathrm {w}}}{\mathbf {J}}_{\mathrm {w}}\nonumber \\&\quad -D_{\mathrm {i}}{\mathbf {C}}^{-1}\left[ \nabla C_{\mathrm {i}}-\frac{C_{\mathrm {i}}}{C_{\mathrm {w}}}\nabla C_{\mathrm {w}}+\frac{F}{{\mathcal {R}}T}z_{\mathrm {i}}C_{\mathrm {i}}\nabla \psi \right] \,, \end{aligned}$$

49c $$\begin{aligned} {\mathbf {J}}_{\mathrm {w}}^{\mathrm {e}}=-D_{\mathrm {w}}^{\mathrm {e}}{\mathbf {C}}^{-1}\Biggl [\frac{v_{\mathrm {w}}C_{\mathrm {w}}^{\mathrm {e}}}{{\mathcal {R}}T}\nabla p_{\mathrm {w}}+\frac{F}{{\mathcal {R}}T}\sum _{\mathrm {i}}z_{\mathrm {i}}C_{\mathrm {i}}^{\mathrm {e}}\nabla \psi ^{\mathrm {e}}\nonumber \\\quad +\frac{\sum _{{\mathrm {j}}\ne {\mathrm{i}}}C_{\mathrm {j}}^{\mathrm {e}}}{C_{\mathrm {w}}^{\mathrm {e}}}\nabla C_{\mathrm {w}}^{\mathrm {e}}\Biggr ]\,, \end{aligned}$$

49d $$\begin{aligned} {\mathbf {J}}_{\mathrm {i}}^{\mathrm {e}}&=\frac{C_{\mathrm {i}}^{\mathrm {e}}}{C_{\mathrm {w}}^{\mathrm {e}}}{\mathbf {J}}_{\mathrm {w}}^{\mathrm {e}}\nonumber \\&\quad -D_{\mathrm {i}}^{\mathrm {e}}{\mathbf {C}}^{-1}\left[ \nabla C_{\mathrm {i}}^{\mathrm {e}}-\frac{C_{\mathrm {i}}^{\mathrm {e}}}{C_{\mathrm {w}}^{\mathrm {e}}}\nabla C_{\mathrm {w}}^{\mathrm {e}}+\frac{F}{{\mathcal {R}}T}z_{\mathrm {i}}C_{\mathrm {i}}^{\mathrm {e}}\nabla \psi ^{\mathrm {e}}\right] \,, \end{aligned}$$

where the index ${\mathrm {i}}$ refers to mobile ions, whereas the index ${\mathrm {j}}$ refers to fixed ions.

In particular, the first terms in Eqs. (49a) and (49c) account for the water flux down its pressure gradient; they arise because of the coupling of water transport with mechanics. The last terms in Eqs. (49b) and (49d) account for migration, that is, the ion transport in an electric field; they result from the coupling of ion transport with electrostatics. The second terms in Eqs. (49a) and (49c) describe the electro-osmosis of water with mobile ions, while the first terms in Eqs. (49b) and (49d) are associated with the convection of ions with water; electro-osmosis and convection originate from the coupling of water and ion transport. In the absence of ions ($C_{\mathrm {i}}=C_{\mathrm {j}}=C_{\mathrm {i}}^{\mathrm {e}}=C_{\mathrm {j}}^{\mathrm {e}}=0$), the water fluxes (49a) and (49c) reduce to Darcy-like fluxes, as in standard poromechanics (Coussy 2004). For immobile water ($D_{\mathrm {w}}=D_{\mathrm {w}}^{\mathrm {e}}=0$), the ion fluxes (49b) and (49d) reduce to standard Nernst–Planck fluxes, describing the electro-diffusion of ions (Rubinstein 1990).

Substituting Eqs. (38), (42), and (47) in Eqs. (22) leads to the following transmembrane fluxes:

50a $$\begin{aligned}&J_{\mathrm {w}}^{\mathrm {m}}=-D_{\mathrm {w}}^{\mathrm {m}}\frac{C_{\mathrm {w}}+C_{\mathrm {w}}^{\mathrm {e}}}{2T^{\mathrm {m}}}\left[ \frac{C_{\mathrm {w}}^{\mathrm {e}}C-C_{\mathrm {w}}C^{\mathrm {e}}}{C_{\mathrm {w}}C_{\mathrm {w}}^{\mathrm {e}}}\right] \,, \end{aligned}$$

50b $$\begin{aligned}&J_{\mathrm {i}}^{\mathrm {m}}=-D_{\mathrm {i}}^{\mathrm {m}}\frac{C_{\mathrm {i}}+C_{\mathrm {i}}^{\mathrm {e}}}{2T^{\mathrm {m}}}\left[ \ln \left( \frac{C_{\mathrm {i}}^{\mathrm {e}}}{C_{\mathrm {i}}}\frac{C_{\mathrm {w}}}{C_{\mathrm {w}}^{\mathrm {e}}}\right) -\frac{Fz_{\mathrm {i}}}{{\mathcal {R}}T}\psi ^{\mathrm {m}}\right] \,, \end{aligned}$$

where

51 $$\begin{aligned} \psi ^{\mathrm {m}}=\psi -\psi ^{\mathrm {e}} \end{aligned}$$

is the membrane potential. Equation (50a) accounts for the transmembrane osmosis through aquaporins, whereas Eq. (50b) accounts for the transmembrane electro-diffusion of ions through ion channels, historically addressed through the Goldman–Hodgkin–Katz flux equation (Hille 1984).

Finally, we note that, in light of Eq. (34), substituting Eqs. (49a) and (50a) into Eq. (2a) provides an equation to be solved for the water pressure $p_{\mathrm {w}}$.

## One-dimensional axisymmetric benchmark

As a representative benchmark, we consider a circular cell cluster, of reference radius $R^{\mathrm {cl}}$, whose innermost circular region, of reference radius $R^{\mathrm {cl}}/2$ and denoted as $\varOmega _{\mathrm {in}}$, is characterized by a transmembrane diffusivity to sodium $D_{\text {Na}^{+}}^{\text {m}}$ ten times larger than the surrounding annular region, denoted as $\varOmega _{\mathrm {out}}$, simulating an overexpression of sodium channels. Given the axial symmetry of the problem, the results only depend on the radial coordinate *R*. We assume plane stress conditions in Eq. (1), and that each cell is circular in the reference configuration, such that, in Eqs. (2) and (3), $A^{\mathrm {c}}/V^{\mathrm {c}}=2/R^{\mathrm {c}}$, with $R^{\mathrm {c}}$ denoting the reference cell radius.

We derive the governing equations for this 1D axisymmetric problem in “Appendix A” and detail their finite element implementation in *Comsol Multiphysics*^®^  in “Appendix B”. After listing the model parameters in Sect. 4.1, we first present the results of the simulation in the absence of both GJs and TJs, in Sect. 4.2. Then, we introduce and comment on the role of GJs in Sect. 4.3. Finally, we further account for TJs in Sect. 4.4.

### Parameters

**Table 1:** Employed model parameters

Name	Symbol	Reference value	Range explored	Unit
Reference cell radius	$R^{\mathrm {c}}$	5		$\mu {\mathrm {m}}$
Membrane thickness	$T^{\mathrm {m}}$	5		${\mathrm {nm}}$
Reference cluster radius	$R^{\mathrm {cl}}$	500		$\mu {\mathrm {m}}$
Initial IC porosity	$\varPhi _0$	0.695		–
Initial EC porosity	$\varPhi _0^{\mathrm {e}}$	0.005		–
Temperature	*T*	310		${\mathrm {K}}$
Water relative permittivity	$\varepsilon _{\mathrm {r}}$	80		–
Water molar volume	$v_{\mathrm {w}}$	18		${\mathrm {cm}}^3{\mathrm{/mol}}$
Membrane relative permittivity	$\varepsilon _{\mathrm {r}}^{\mathrm {m}}$	3		–
TJ thickness	$T^{\mathrm {tj}}$	500		${\mathrm {nm}}$
TJ relative permittivity	$\varepsilon _{\mathrm {r}}^{\mathrm {tj}}$	30		–
Young modulus	*E*	0.4	$0.4\div 4000$	${\mathrm {kPa}}$
Poisson ratio	$\nu$	0.3		–
Initial IC ${\mathrm {Na}}^{+}$ concentration	$C_{\mathrm {{Na}}^{+}}^0$	10		${\mathrm {mol/m}}^3$
Initial EC ${\mathrm {Na}}^{+}$ concentration	$C_{\mathrm {{Na}}^{+}}^{\mathrm {e,0}}$	145		${\mathrm {mol/m}}^3$
Initial IC ${\mathrm {K}}^{+}$ concentration	$C_{\mathrm {{K}}^{+}}^0$	140		${\mathrm {mol/m}}^3$
Initial EC ${\mathrm {K}}^{+}$ concentration	$C_{\mathrm {{K}}^{+}}^{\mathrm {e,0}}$	5		${\mathrm {mol/m}}^3$
Initial IC ${\mathrm {Cl}}^{-}$ concentration	$C_{\mathrm {{Cl}}^{-}}^0$	10		${\mathrm {mol/m}}^3$
Initial EC ${\mathrm {Cl}}^{-}$ concentration	$C_{\mathrm {{Cl}}^{-}}^{\mathrm {e,0}}$	110		${\mathrm {mol/m}}^3$
IC fixed anion concentration	$C_{\mathrm {A}}^{-}$	140		${\mathrm {mol/m}}^3$
EC fixed anion concentration	$C_{\mathrm {{A}}^{-}}^{\mathrm {e}}$	40		${\mathrm {mol/m}}^3$
Transmembrane ${\mathrm {Na}}^{+}$ diffusivity	$D_{\mathrm {{Na}}^{+}}^{\mathrm {m}}$	$10^{-18}\,(\varOmega _{\mathrm {out}})$		${\mathrm {m}}^2/{\mathrm{s}}$
		$10^{-17}\,(\varOmega _{\mathrm {in}})$		${\mathrm {m}}^2/{\mathrm{s}}$
Transmembrane ${\mathrm {{K}}^{+}}$ diffusivity	$D_{\mathrm {{K}}^{+}}^{\mathrm {m}}$	$5\times 10^{-18}$		${\mathrm {m}}^2/{\mathrm{s}}$
Transmembrane ${\mathrm {{Cl}}^{-}}$ diffusivity	$D_{\mathrm {{Cl}}^{-}}^{\mathrm {m}}$	$5\times 10^{-17}$		${\mathrm {m}}^2/{\mathrm{s}}$
Transmembrane water diffusivity	$D_{\mathrm {w}}^{\mathrm {m}}$	$10^{-8}$	$10^{-14}\div 10^{-8}$	${\mathrm {m}}^2/{\mathrm{s}}$
EC ${\mathrm {Na}}^{+}$, ${\mathrm {K}}^{+}$, and ${\mathrm {Cl}}^{-}$ diffusivity	$D_{\mathrm {i}}^{\mathrm {e}}$	$10^{-9}$		${\mathrm {m}}^2/{\mathrm{s}}$
EC water diffusivity	$D_{\mathrm {w}}^{\mathrm {e}}$	$10^{-7}$	$10^{-8}\div 10^{-6}$	${\mathrm {m}}^2/{\mathrm{s}}$
IC ${\mathrm {Na}}^{+}$, ${\mathrm {K}}^{+}$, and ${\mathrm {Cl}}^{-}$ diffusivity (with GJs)	$D_{\mathrm {i}}$	$10^{-12}$		${\mathrm {m}}^2/{\mathrm{s}}$
IC water diffusivity (with GJs)	$D_{\mathrm {w}}$	$10^{-9}$	$10^{-10}\div 10^{-8}$	${\mathrm {m}}^2/{\mathrm{s}}$

The model parameters are listed in Table 1. We refer to an average animal cell of reference radius $R^{\mathrm {c}}=5\,\mu {\mathrm {m}}$ and membrane thickness $T^{\mathrm {m}}=5\,{\mathrm {nm}}$. The cluster reference radius is $R^{\mathrm {cl}}=500\,\mu {\mathrm {m}}$, which is much larger than $R^{\mathrm {c}}$, that is, the characteristic size of a material point, thus ensuring the validity of our continuum formulation. We assume that cells are separated by a reference intercellular space of about $30\,{\mathrm {nm}}$ in size (Pietak and Levin 2016), and that approximately the 70% of the cluster is occupied by fluid. Correspondingly, we obtain an estimate of $\varPhi _0=0.695$ and $\varPhi _0^{\mathrm {e}}=0.005$ for the initial IC and EC porosities.

The simulations are conducted at body temperature $T=310\,{\mathrm {K}}$. Accordingly, $\varepsilon _{\mathrm {r}}=80$ and $v_{\mathrm {w}}=18\,{\mathrm{cm}}^3{\mathrm{/mol}}$ are reliable estimates for the relative permittivity and molar volume of water. We employ $\varepsilon _{\mathrm {r}}^{\mathrm {m}}=3$ for the relative permittivity of the cell membrane (Gramse et al. 2013). The thickness of a TJ complex is about $T^\mathrm {tj}=500\,{\mathrm {nm}}$ (Tsukita et al. 2001), and we use $\varepsilon _{\mathrm {r}}^{\mathrm {tj}}=30$ for its relative permittivity, which is an average between those of bulk water and proteins inside (Li et al. 2013).

We choose a representative value $E=0.4\,{\mathrm {kPa}}$ for the Young modulus and assume a Poisson ratio $\nu =0.3$ (Moeendarbary et al. 2013). The Lamé parameters entering Eq. (24) thus read $\lambda =E\nu /[(1+\nu )(1-2\nu )]\approx 0.23\,{\mathrm {kPa}}$ and $G=E/[2(1+\nu )]\approx 0.15\,{\mathrm {kPa}}$. In Sect. 4.3, we also consider the case of a larger *E*, simulating a cluster of plant cells, equipped with stiff cell walls.

With reference to a typical mammalian cell, the more abundant ions involved in bioelectricity are sodium, potassium, and chloride. We adopt the following initial IC and EC concentrations (Alberts 1983): $C_{\mathrm {{Na}}^{+}}^0=10\,{\mathrm {mol/m}}^3$, $C_{\mathrm {{Na}}^{+}}^{\mathrm {{e}},0}=145\,{\mathrm {mol/m}}^3$, $C_{\mathrm {{K}}^{+}}^0=140\,{\mathrm {mol/m}}^3$, $C_{\mathrm {{K}}^{+}}^{\mathrm {{e}},0}=5\,{\mathrm {mol/m}}^3$, $C_{\mathrm {{Cl}}^{-}}^0=10\,{\mathrm {mol/m}}^3$, and $C_{\mathrm {{Cl}}^{-}}^{\mathrm {{e}},0}=110\,{\mathrm {mol/m}}^3$. We also consider a fixed generic monovalent anion, whose IC and EC concentrations are uniform and constant and equal to $C_{\mathrm {{A}}^{-}}=140\,{\mathrm {mol/m}}^3$ and $C_{\mathrm {{A}}^{-}}^{\mathrm {e}}=40\,{\mathrm {mol/m}}^3$. In the IC space, ${\mathrm {A}}^-$ is intended to represent negatively charged proteins, nucleic acids, and other cellular constituents. Notably, $C_{\mathrm {{A}}^-}$ and $C_{\mathrm {{A}}^-}^{\mathrm {e}}$ ensure the initial electroneutrality in both the IC and EC spaces (that is, $\sum _{\mathrm {i}}z_{\mathrm {i}}C_{\mathrm {i}}^0=\sum _{\mathrm {i}}z_{\mathrm {i}}C_{\mathrm {i}}^{\mathrm {e,0}}=0$) and also the equality of the initial IC and EC osmotic concentrations (that is, $C^0=C^{\mathrm {e,0}}=300\,{\mathrm {mol/m}}^3$). By using Eq. (31), we obtain $C_{\mathrm {w}}^0=C_{\mathrm {w}}^{\mathrm {e,0}}=1/v_{\mathrm {w}}\approx 5.6\times 10^4\,{\mathrm {mol/m}}^3$. Therefore, the IC and EC solutions are actually dilute; indeed, $C^0/C_{\mathrm {w}}^0=C^{\mathrm {e,0}}/C_{\mathrm {w}}^{\mathrm {e,0}}\approx 0.5\%$.

We employ the following transmembrane ion diffusivities: $D_{\mathrm {{Na}}^{+}}^{\mathrm {m}}=10^{-18}\,{\mathrm {m}}^2/{\mathrm{s}}$, $D_{\mathrm {{K}}^{+}}^{\mathrm {m}}=5\times 10^{-18}\,{\mathrm {m}}^2/{\mathrm {s}}$, and $D_{\mathrm {{Cl}}^{-}}^{\mathrm {m}}=5\times 10^{-17}\,{\mathrm {m}}^2/{\mathrm {s}}$. These are on the order of those reported in Pietak and Levin (2016), but account for the fact that the permeability of artificial lipid bilayers to ${\mathrm {{Na}}^{+}}$, ${\mathrm {{K}}^{+}}$, and ${\mathrm {{Cl}}^{-}}$ is not the same (Alberts 1983). As anticipated, in $\varOmega _{\mathrm {in}}$ we set instead $D_{\mathrm {{Na}}^{+}}^{\mathrm {m}}=10^{-17}\,{\mathrm {m}}^2/{\mathrm {s}}$. We consider that the transmembrane water diffusivity is $D_{\mathrm {w}}^{\mathrm {m}}=10^{-8}\,{\mathrm {m}}^2/{\mathrm {s}}$, that is, ten order of magnitudes larger than $D_{\mathrm {{Na}}^{+}}^{\mathrm {m}}$, as documented in Alberts (1983) with reference to artificial lipid bilayers. In Sect. 4.2, we further analyze the case of smaller $D_{\mathrm {w}}^{\mathrm {m}}$, simulating an underexpression of aquaporins.

We follow Pietak and Levin (2016) and assume $D_{\mathrm {i}}^{\mathrm {e}}=10^{-9}\,{\mathrm {m}}^2/{\mathrm {s}}$ for the diffusivity of all ions in EC water. We set $D_{\mathrm {w}}^{\mathrm {e}}=10^{-7}\,{\mathrm {m}}^2/{\mathrm {s}}$ for the EC water diffusivity, as approximately obtained through the Kozeny–Carman equation (Coussy 2004). Given the uncertainty in this parameter, in Sect. 4.2 we also explore how the response changes by increasing or decreasing $D_{\mathrm {w}}^{\mathrm {e}}$ of one order of magnitude.

In the presence of GJs, we adopt $D_{\mathrm {i}}=10^{-12}\,{\mathrm {m}}^2/{\mathrm{s}}$ for the diffusivity of all ions in IC water. In particular, $D_{\mathrm {i}}\le 10^{-14}\,{\mathrm {m}}^2/{\mathrm{s}}$ should be excluded, as it has no impact on the behavior of the cluster. Finally, we adopt $D_{\mathrm {w}}=10^{-9}\,{\mathrm {m}}^2/{\mathrm{s}}$ for the IC water diffusivity and, in Sect. 4.3, we further explore how the cluster behavior is affected by variations of $D_{\mathrm {w}}$ of one order of magnitude.

### Results in the absence of gap and tight junctions

**Fig. 2:**
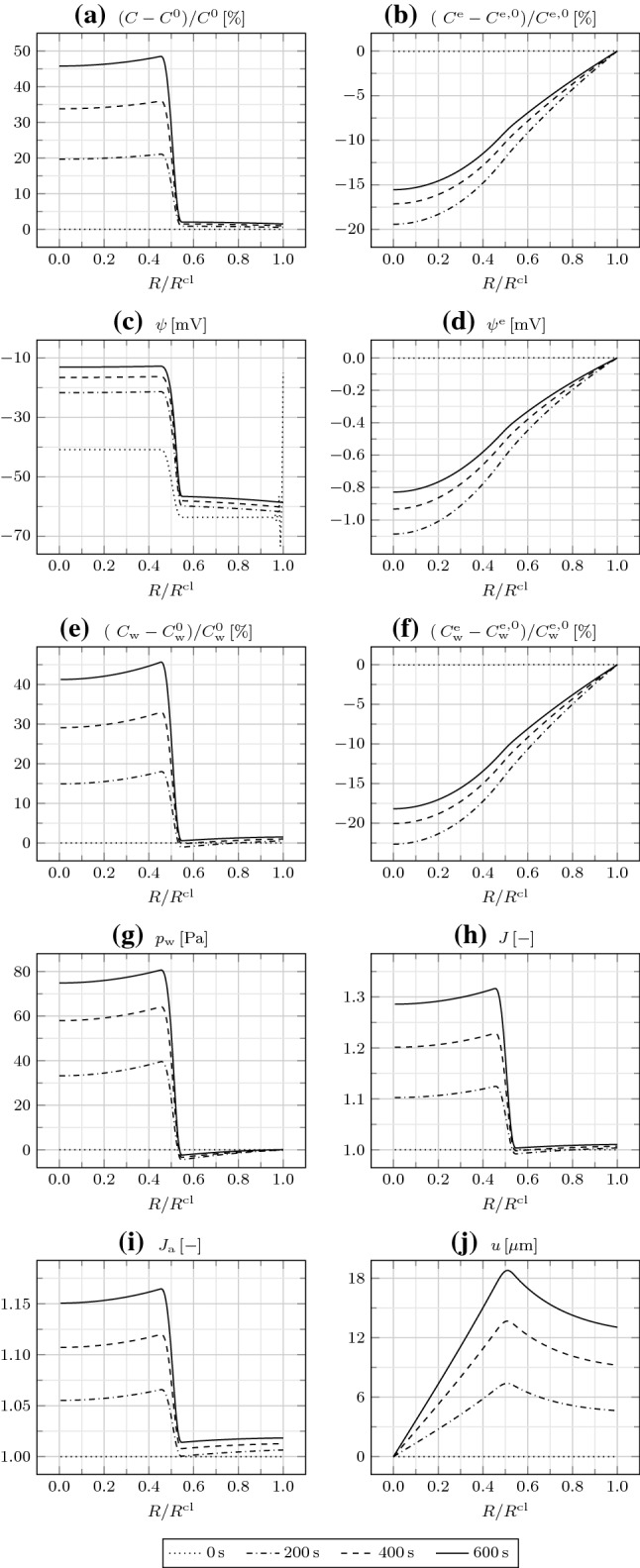
**a** Relative IC osmotic concentration $(C-C^0)/C^0$, **b** relative EC osmotic concentration $(C^{\mathrm {e}}-C^{\mathrm {e,0}})/C^{\mathrm {e,0}}$, **c** IC electric potential $\psi$, **d** EC electric potential $\psi ^{\mathrm {e}}$, **e** relative IC water concentration $(C_{\mathrm {w}}-C_{\mathrm {w}}^0)/C_{\mathrm {w}}^0$, **f** relative EC water concentration $(C_{\mathrm {w}}^{\mathrm {e}}-C_{\mathrm {w}}^{\mathrm {e,0}})/C_{\mathrm {w}}^{\mathrm {e,0}}$, **g** water pressure $p_{\mathrm {w}}$, **h** Jacobian *J*, **i** areal Jacobian $J_{\mathrm {a}}$, and **j** radial displacement *u* as a function of $R/R^{\mathrm {cl}}$ at different times

We first assume that GJs are either absent or closed, such that $D_{\mathrm {w}}=D_{\mathrm {i}}=0$ in Eqs. (49a) and (49b). Therefore, the mass balances (2a) and (3a) reduce to ordinary differential equations. Moreover, we assume that TJs are absent, such that boundary conditions (8) and (11) hold. In Fig. 2, we represent the relevant bioelectrical and mechanical fields as a function of *R* at different times.

The large $D_{\mathrm {{Na}}^{+}}^{\mathrm {m}}$ in $\varOmega _{\mathrm {in}}$ leads to a prominent influx of ${\mathrm {Na}}^+$ from the EC to the IC space, that is, down its concentration gradient. Correspondingly, the IC osmotic concentration *C* rapidly increases. We register a little increase in *C* in $\varOmega _{\mathrm {out}}$ as well, which is essentially due to the large $C_{\mathrm {{A}}^{-}}$ compared to $C_{\mathrm {{A}}^{-}}^{\mathrm {e}}$, as explained by the Gibbs–Donnan effect (Overbeek 1956). While *C* presents a steep gradient at $R=R^{\mathrm {cl/2}}$, due to the lack of GJs connecting $\varOmega _{\mathrm {in}}$ and $\varOmega _{\mathrm {out}}$, the EC osmotic concentration $C^{\mathrm {e}}$ is smoother, because of the interconnection of the intercellular spaces. Moreover, while $C^{\mathrm {e}}$ initially decreases with time, it then increases as ${\mathrm {Na}}^+$ is transported from the outside to the inside of the cluster.

The redistribution of ions establishes a negative IC electric potential $\psi$, as of the beginning of the simulation. This is again explained by the Gibbs–Donnan effect. In particular, $\varOmega _{\mathrm {in}}$ is depolarized, that is, at a higher $\psi$, with respect to $\varOmega _{\mathrm {out}}$, and the depolarization increases over time due to the influx of ${\mathrm {Na}}^+$. The EC electric potential $\psi ^{\mathrm {e}}$ remains rather small, such that the membrane potential $\psi ^{\mathrm {m}}$ nearly corresponds to $\psi$. In particular, the value of about $-60\,{\mathrm {mV}}$, registered in $\varOmega _{\mathrm {out}}$, is representative of the resting $\psi ^{\mathrm {m}}$ associated with the adopted initial ion concentrations and transmembrane diffusivities, which can be estimated through the Goldman–Hodgkin–Katz voltage equation (Hille 1984).

As ${\mathrm {Na}}^+$ is transported from the EC to the IC space through ion channels in $\varOmega _{\mathrm {in}}$, water follows by osmosis through aquaporins. Correspondingly, the IC water concentration $C_{\mathrm {w}}$ increases with time. In the EC space, as ${\mathrm {Na}}^+$ ions enter the cluster to cope with the request for ${\mathrm {Na}}^+$ in $\varOmega _{\mathrm {in}}$, they drag water molecules by electro-osmosis. Therefore, the EC water concentration $C_{\mathrm {w}}^{\mathrm {e}}$ increases after initially decreasing, similar to $C^{\mathrm {e}}$.

As water enters $\varOmega _{\mathrm {in}}$, the water pressure $p_{\mathrm {w}}$ increases therein and is equilibrated by the mechanical stress. The IC and EC electrostatic pressures $p_{\mathrm {pol}}$ and $p_{\mathrm {pol}}^{\mathrm {e}}$, not represented here, are both irrelevant, being orders of magnitude lower than $p_{\mathrm {w}}$. The increase in $C_{\mathrm {w}}$ in $\varOmega _{\mathrm {in}}$ is also accompanied by an increase in the Jacobian *J*. We remark that, given the smaller variation of $C_{\mathrm {w}}^{\mathrm {e}}$ compared to $C_{\mathrm {w}}$ except for the initial transient, and, mostly, the close cell packing, implying $\varPhi _0\gg \varPhi _0^{\mathrm {e}}$, the contribution of the variation of $C_{\mathrm {w}}^{\mathrm {e}}$ to *J*, as described by Eq. (30), is negligible.

The areal Jacobian $J_{\mathrm {a}}$, given by the product of the radial and circumferential deformation gradient components (or stretches) $F_{rR}$ and $F_{\theta \varTheta }$, indicates in-plane expansion everywhere, larger in $\varOmega _{\mathrm {in}}$. By comparing *J* and $J_{\mathrm {a}}$, we infer that there occurs an out-of-plane expansion in $\varOmega _{\mathrm {in}}$ and a little out-of-plane compression in $\varOmega _{\mathrm {out}}$. Finally, the radial displacement *u* increases from $R=0$ to $R=R^{\mathrm {cl}}/2$, and then decreases.

**Fig. 3:**
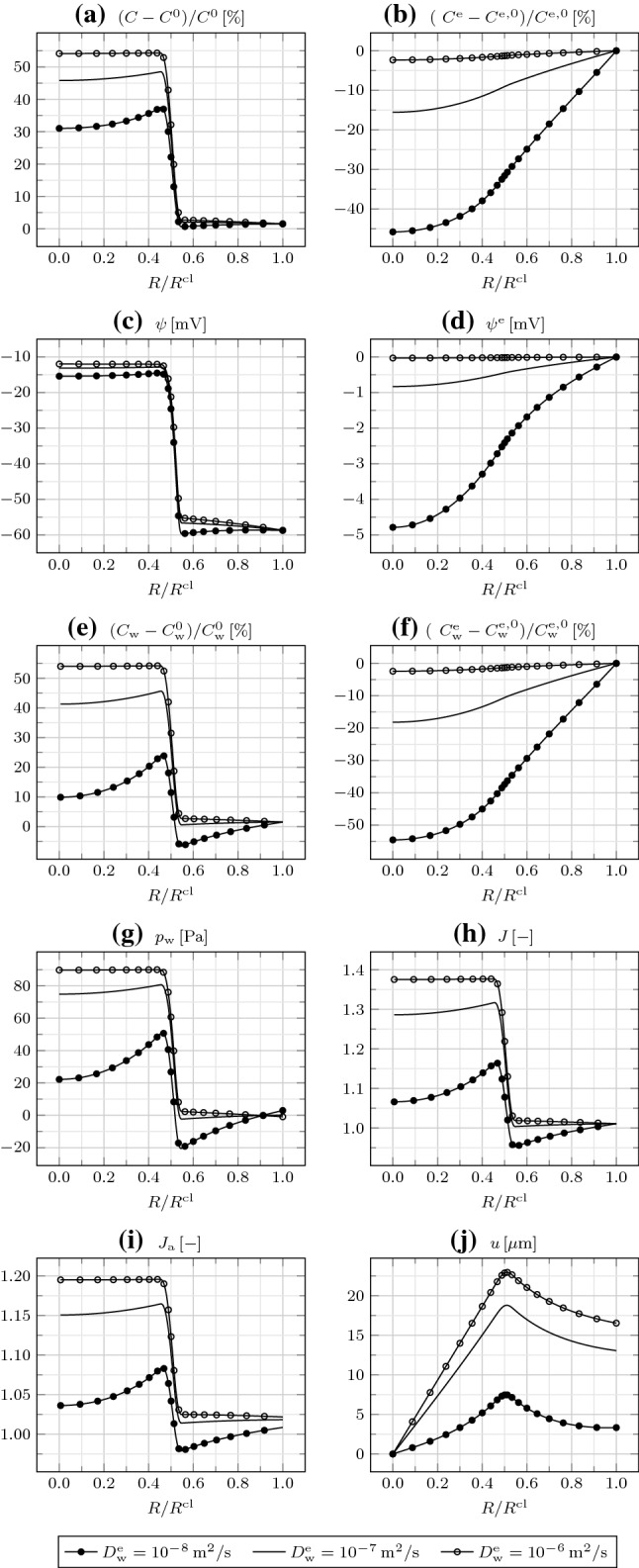
**a** Relative IC osmotic concentration $(C-C^0)/C^0$, **b** relative EC osmotic concentration $(C^{\mathrm {e}}-C^{\mathrm {e,0}})/C^{\mathrm {e,0}}$, **c** IC electric potential $\psi$, **d** EC electric potential $\psi ^{\mathrm {e}}$, **e** relative IC water concentration $(C_{\mathrm {w}}-C_{\mathrm {w}}^0)/C_{\mathrm {w}}^0$, **f** relative EC water concentration $(C_{\mathrm {w}}^{\mathrm {e}}-C_{\mathrm {w}}^{\mathrm {e,0}})/C_{\mathrm {w}}^{\mathrm {e,0}}$, **g** water pressure $p_{\mathrm {w}}$, **h** Jacobian *J*, **i** areal Jacobian $J_{\mathrm {a}}$, and **j** radial displacement *u* as a function of $R/R^{\mathrm {cl}}$ at $t=600\,{\mathrm {s}}$ for different EC water diffusivities $D_{\mathrm {w}}^{\mathrm {e}}$

In Fig. 3, we explore how the same fields are affected by variations of the EC water diffusivity $D_{\mathrm {w}}^{\mathrm {e}}$. By increasing $D_{\mathrm {w}}^{\mathrm {e}}$, water is transported more rapidly from the outside to the inside of the cluster through the EC space, and consequently from the EC to the IC space across cell membranes. Therefore, fixed the time, $C_{\mathrm {w}}$, $C_{\mathrm {w}}^{\mathrm {e}}$, $p_{\mathrm {w}}$, *J*, $J_{\mathrm {a}}$, and *u* increase. Interestingly, *C* and $C^{\mathrm {e}}$ increase as well, since the convective contribution to ion transport grows with $D_{\mathrm {w}}^{\mathrm {e}}$. The change in the ion redistribution also impacts on $\psi ^{\mathrm {m}}$, though mildly. Finally, we note that, by decreasing $D_{\mathrm {w}}^{\mathrm {e}}$ to $10^{-8}\,{\mathrm {m}}^2/{\mathrm{s}}$, the demand for water in $\varOmega _{\mathrm {in}}$ can be barely sustained, such that, for a given time, $C_{\mathrm {w}}$ decreases from $R=R^{\mathrm {cl}}/2$ to $R=0$; furthermore, $C_{\mathrm {w}}$ also decreases from $R=R^{\mathrm {cl}}$ to $R=R^{\mathrm {cl}}/2$, suggesting that water is transported from the IC to the EC space in $\varOmega _{\mathrm {out}}$, and then from $\varOmega _{\mathrm {out}}$ to $\varOmega _{\mathrm {in}}$ through the EC space, resulting in an in-plane shrinkage of $\varOmega _{\mathrm {out}}$.

**Fig. 4:**
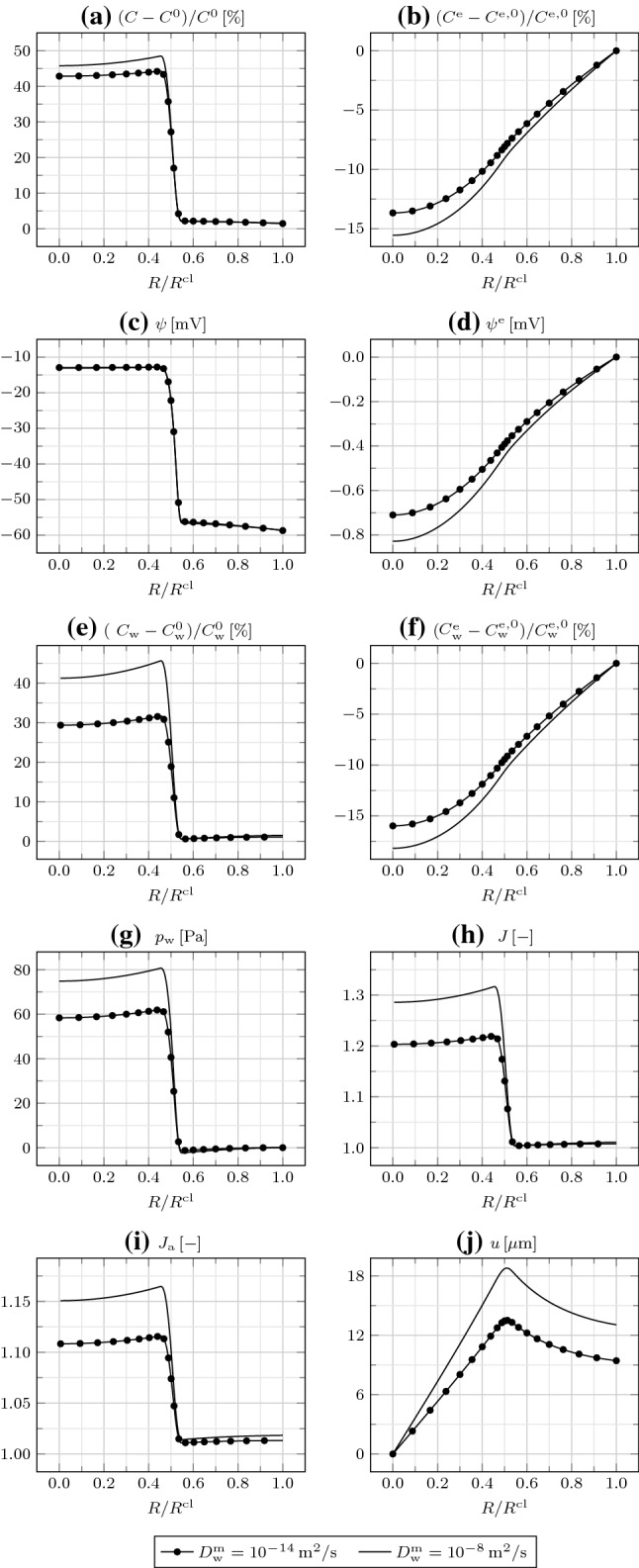
**a** Relative IC osmotic concentration $(C-C^0)/C^0$, **b** relative EC osmotic concentration $(C^{\mathrm {e}}-C^{\mathrm {e,0}})/C^{\mathrm {e,0}}$, **c** IC electric potential $\psi$, **d** EC electric potential $\psi ^{\mathrm {e}}$, **e** relative IC water concentration $(C_{\mathrm {w}}-C_{\mathrm {w}}^0)/C_{\mathrm {w}}^0$, **f** relative EC water concentration $(C_{\mathrm {w}}^{\mathrm {e}}-C_{\mathrm {w}}^\mathrm {e,0})/C_{\mathrm {w}}^{\mathrm {e,0}}$, **g** water pressure $p_{\mathrm {w}}$, **h** Jacobian *J*, **i** areal Jacobian $J_{\mathrm {a}}$, and **j** radial displacement *u* as a function of $R/R^{\mathrm {cl}}$ at $t=600\,{\mathrm {s}}$ for different transmembrane water diffusivities $D_{\mathrm {w}}^{\mathrm {m}}$

In Fig. 4, we consider the effect of the transmembrane water diffusivity $D_{\mathrm {w}}^{\mathrm {m}}$, modulated by the density and open fraction of aquaporins. Increasing $D_{\mathrm {w}}^{\mathrm {m}}$ above $10^{-8}\,{\mathrm {m}}^2/{\mathrm{s}}$ or decreasing it up to $10^{-12}\,{\mathrm {m}}^2/{\mathrm{s}}$ does not affect the results. By further decreasing $D_{\mathrm {w}}^{\mathrm {m}}$ to $10^{-14}\,{\mathrm {m}}^2/{\mathrm{s}}$, the transmembrane water transport is hampered, such that $C_{\mathrm {w}}$ strongly decreases and $C_{\mathrm {w}}^{\mathrm {e}}$ increases. In turn, this determines a decrease in $p_{\mathrm {w}}$, *J*, $J_{\mathrm {a}}$, and *u*. Importantly, while varying $D_{\mathrm {w}}^{\mathrm {e}}$ strongly impacted on the ion redistribution, changing $D_{\mathrm {w}}^{\mathrm {m}}$ mildly affects it. Indeed, $D_{\mathrm {w}}^{\mathrm {e}}$ enters the EC ion fluxes through the convective contribution [see Eqs. (49c) and (49d)], while $D_{\mathrm {w}}^{\mathrm {m}}$ does not govern the transmembrane ion fluxes, given that aquaporins and ion channels are specific for water and ions [see Eqs. (50)]. The impact of $D_{\mathrm {w}}^{\mathrm {m}}$ on $\psi ^{\mathrm {m}}$ is also negligible.

### Introducing gap junctions

**Fig. 5:**
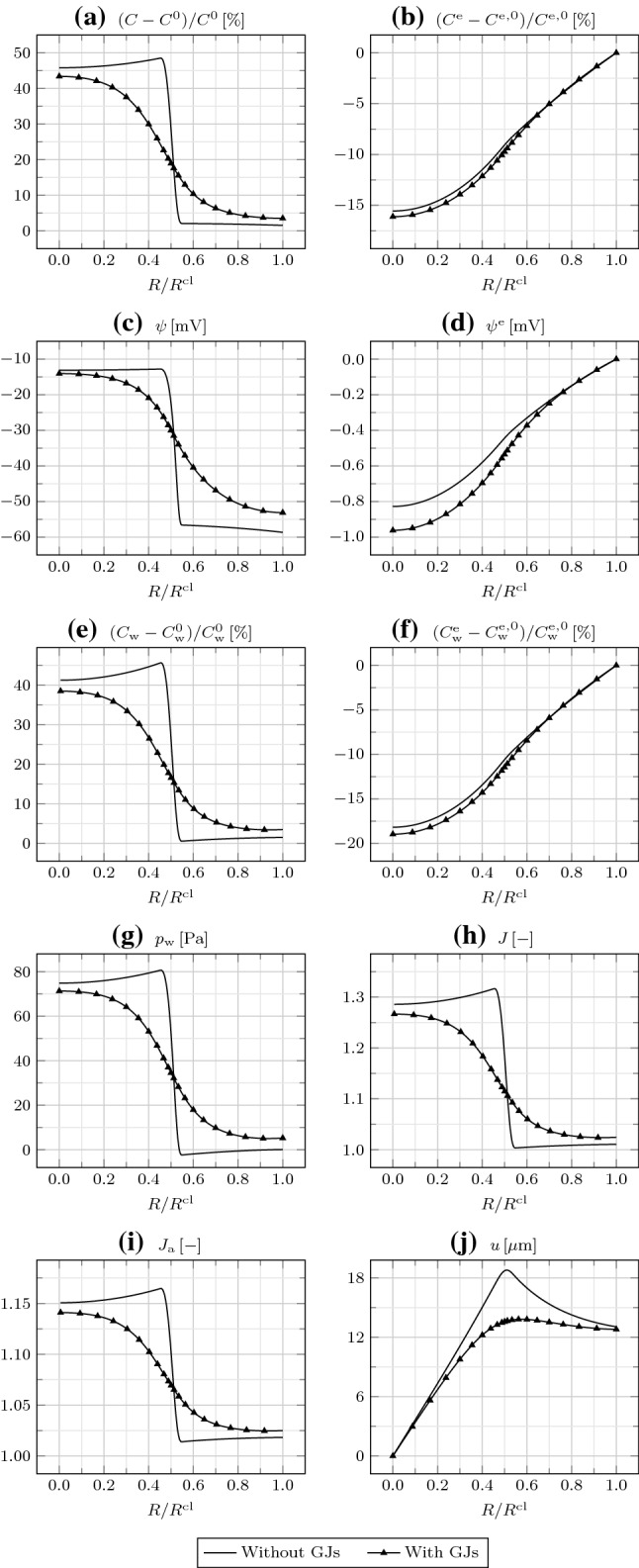
**a** Relative IC osmotic concentration $(C-C^0)/C^0$, **b** relative EC osmotic concentration $(C^{\mathrm {e}}-C^{\mathrm {e,0}})/C^{\mathrm {e,0}}$, **c** IC electric potential $\psi$, **d** EC electric potential $\psi ^{\mathrm {e}}$, **e** relative IC water concentration $(C_{\mathrm {w}}-C_{\mathrm {w}}^0)/C_{\mathrm {w}}^0$, **f** relative EC water concentration $(C_{\mathrm {w}}^{\mathrm {e}}-C_{\mathrm {w}}^{\mathrm {{e}},0})/C_{\mathrm {w}}^{\mathrm {{e}},0}$, **g** water pressure $p_{\mathrm {w}}$, **h** Jacobian *J*, **i** areal Jacobian $J_{\mathrm {a}}$, and **j** radial displacement *u* as a function of $R/R^{\mathrm {cl}}$ at $t=600\,{\mathrm {s}}$ without and with gap junctions

We now investigate on the role of GJs on the mechanobioelectricity of the cluster. As reported in Sect. 4.1, we adopt $D_{\mathrm {i}}=10^{-12}\,{\mathrm {m}}^2/{\mathrm{s}}$ uniformly for all ions and $D_{\mathrm {w}}=10^{-9}\,{\mathrm {m}}^2/{\mathrm{s}}$.

In Fig. 5, we compare the relevant fields at the end of the simulation with and without GJs. If GJs are present, the ${\mathrm {Na}}^+$ ions entering the IC space in $\varOmega _{\mathrm {in}}$ flow down their IC electrochemical potential gradient toward $\varOmega _{\mathrm {out}}$. Therefore, accounting for GJs smooths out the steep gradient of *C* at $R=R^{\mathrm {cl}}/2$, thus leading to a reduction in *C* in $\varOmega _{\mathrm {in}}$ and to an increase in *C* in $\varOmega _{\mathrm {out}}$. The different ion redistribution in the IC space also influences $\psi$, with a lesser depolarization occurring in $\varOmega _{\mathrm {in}}$ and a larger one characterizing $\varOmega _{\mathrm {out}}$. Similarly, the water entering the IC space in $\varOmega _{\mathrm {in}}$ flows toward $\varOmega _{\mathrm {out}}$ through GJs, mainly dragged by ions through electro-osmosis. Predictably, the EC fields are almost no affected by GJs. Notably, the IC water redistribution in the presence of GJs leads to a decrease in $J_{\mathrm {a}}$ in $\varOmega _{\mathrm {in}}$ and to an increase in $J_{\mathrm {a}}$ in $\varOmega _{\mathrm {out}}$; correspondingly, $u(R^{\mathrm {cl}}/2)$ diminishes, but $u(R^{\mathrm {cl}})$ remains equal.

**Fig. 6:**
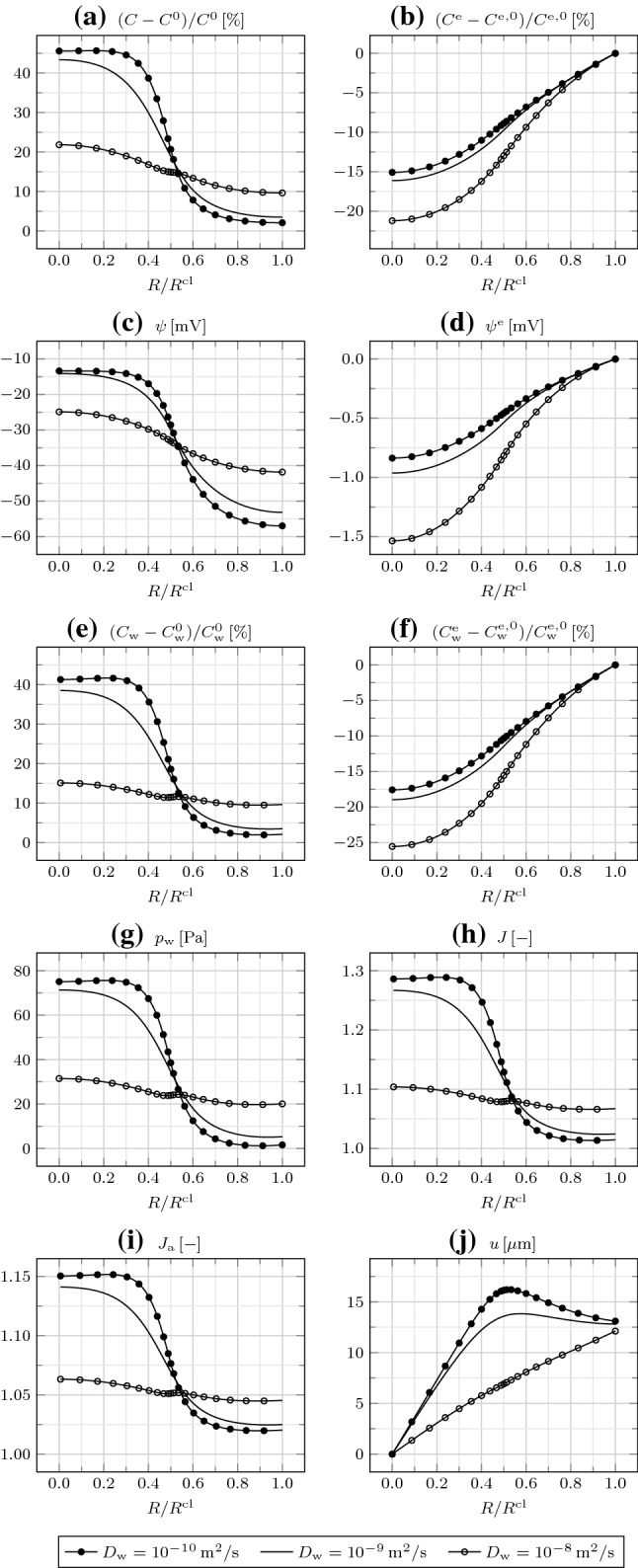
**a** Relative IC osmotic concentration $(C-C^0)/C^0$, **b** relative EC osmotic concentration $(C^{\mathrm {e}}-C^{\mathrm {e,0}})/C^{\mathrm {e,0}}$, **c** IC electric potential $\psi$, **d** EC electric potential $\psi ^{\mathrm {e}}$, **e** relative IC water concentration $(C_{\mathrm {w}}-C_{\mathrm {w}}^0)/C_{\mathrm {w}}^0$, **f** relative EC water concentration $(C_{\mathrm {w}}^{\mathrm {e}}-C_{\mathrm {w}}^{\mathrm {e,0}})/C_{\mathrm {w}}^{\mathrm {e,0}}$, **g** water pressure $p_{\mathrm {w}}$, **h** Jacobian *J*, **i** areal Jacobian $J_{\mathrm {a}}$, and **j** radial displacement *u* as a function of $R/R^{\mathrm {cl}}$ at $t=600\,{\mathrm {s}}$ for different IC water diffusivities $D_{\mathrm {w}}$

In Fig. 6, we compare the responses for different values of $D_{\mathrm {w}}$. By increasing $D_{\mathrm {w}}$, more water is transported from $\varOmega _{\mathrm {in}}$ to $\varOmega _\mathrm {out}$, such that the difference in $C_{\mathrm {w}}$ between $\varOmega _{\mathrm {in}}$ and $\varOmega _\mathrm {out}$ reduces, along with that in $J_{\mathrm {a}}$. However, again, $u(R^{\mathrm {cl}})$ remains the same. The difference in *C* between $\varOmega _{\mathrm {in}}$ and $\varOmega _{\mathrm {out}}$ reduces as well, which confirms the relevance of ion transport by convection as $D_{\mathrm {w}}$ is risen. Decreasing $D_{\mathrm {w}}$ below $10^{-10}\,{\mathrm {m}}^2/{\mathrm{s}}$ does not affect further the results.

**Fig. 7:**
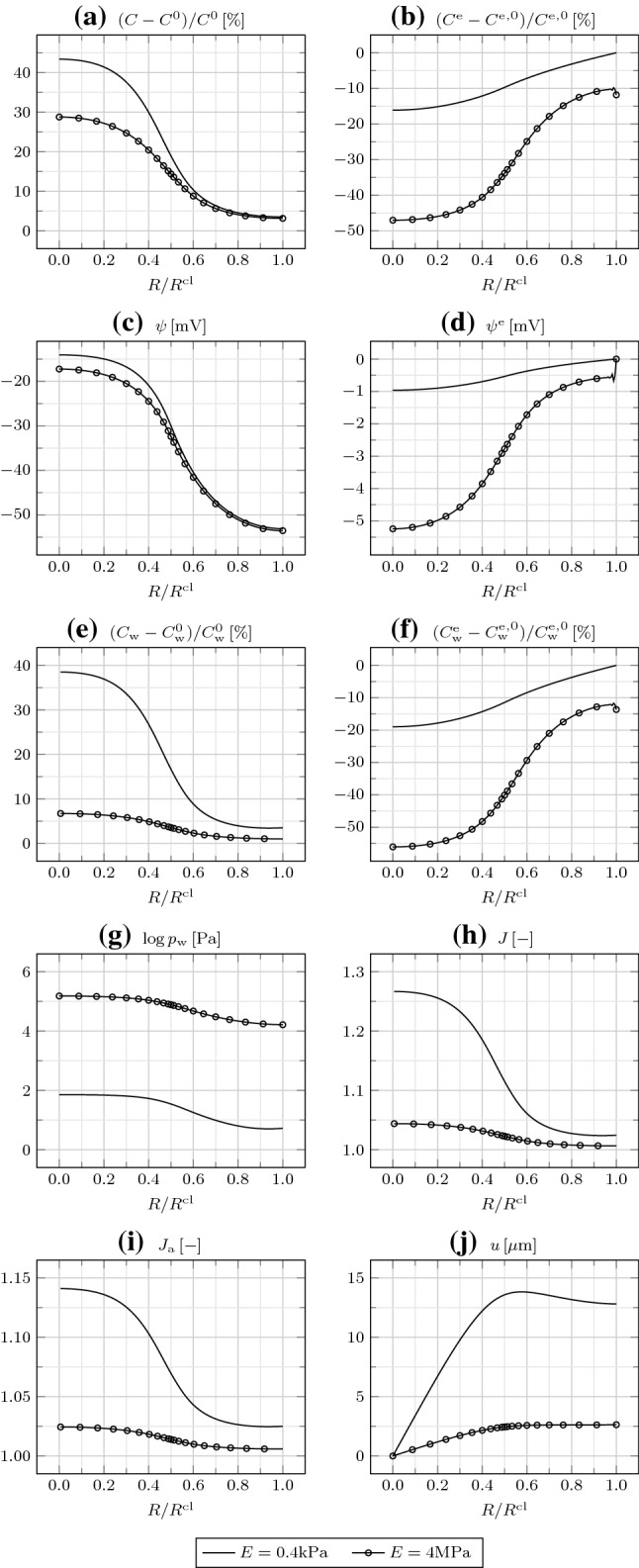
**a** Relative IC osmotic concentration $(C-C^0)/C^0$, **b** relative EC osmotic concentration $(C^{\mathrm {e}}-C^{\mathrm {e,0}})/C^{\mathrm {e,0}}$, **c** IC electric potential $\psi$, **d** EC electric potential $\psi ^{\mathrm {e}}$, **e** relative IC water concentration $(C_{\mathrm {w}}-C_{\mathrm {w}}^0)/C_{\mathrm {w}}^0$, **f** relative EC water concentration $(C_{\mathrm {w}}^{\mathrm {e}}-C_{\mathrm {w}}^{\mathrm {e,0}})/C_{\mathrm {w}}^{\mathrm {e,0}}$, **g** water pressure $p_{\mathrm {w}}$, **h** Jacobian *J*, **i** areal Jacobian $J_{\mathrm {a}}$, and **j** radial displacement *u* as a function of $R/R^{\mathrm {cl}}$ at $t=600\,{\mathrm {s}}$ for different Young moduli *E*, in the presence of gap junctions

In Fig. 7, we examine the cluster behavior by varying its Young modulus *E*. Increasing it up to one order of magnitude does not affect the results, except for $p_{\mathrm {w}}$, which grows proportionally to *E*. Therefore, we conclude that, for suitably small values of *E*, proper of animal cells, the response of the cluster to the imposed bioelectrical perturbation is independent of *E*. More specifically, the ion redistribution triggers the water redistribution, which establishes the cluster deformation. However, for larger values of *E*, which may be proper of plant cells endowed with stiff walls, the mechanics affects the water redistribution as well. Indeed, the accumulation of water in the IC space of $\varOmega _{\mathrm {in}}$ determines the build-up of a large water (turgor) pressure gradient $\nabla p_{\mathrm {w}}$, forcing water to flow back toward $\varOmega _{\mathrm {out}}$, through both GJs and the EC space according to our model [see Eqs. (49a) and (49c)]. Consequently, $C_{\mathrm {w}}$ and $C_{\mathrm {w}}^{\mathrm {e}}$ both diminish, along with $J_{\mathrm {a}}$ and *u*. Though at a lesser extent, *C* and $C^{\mathrm {e}}$ are affected as well, while $\psi ^{\mathrm {m}}$ practically remains unaltered, given the similar reductions in both $\psi$ and $\psi ^{\mathrm {e}}$.

**Fig. 8:**
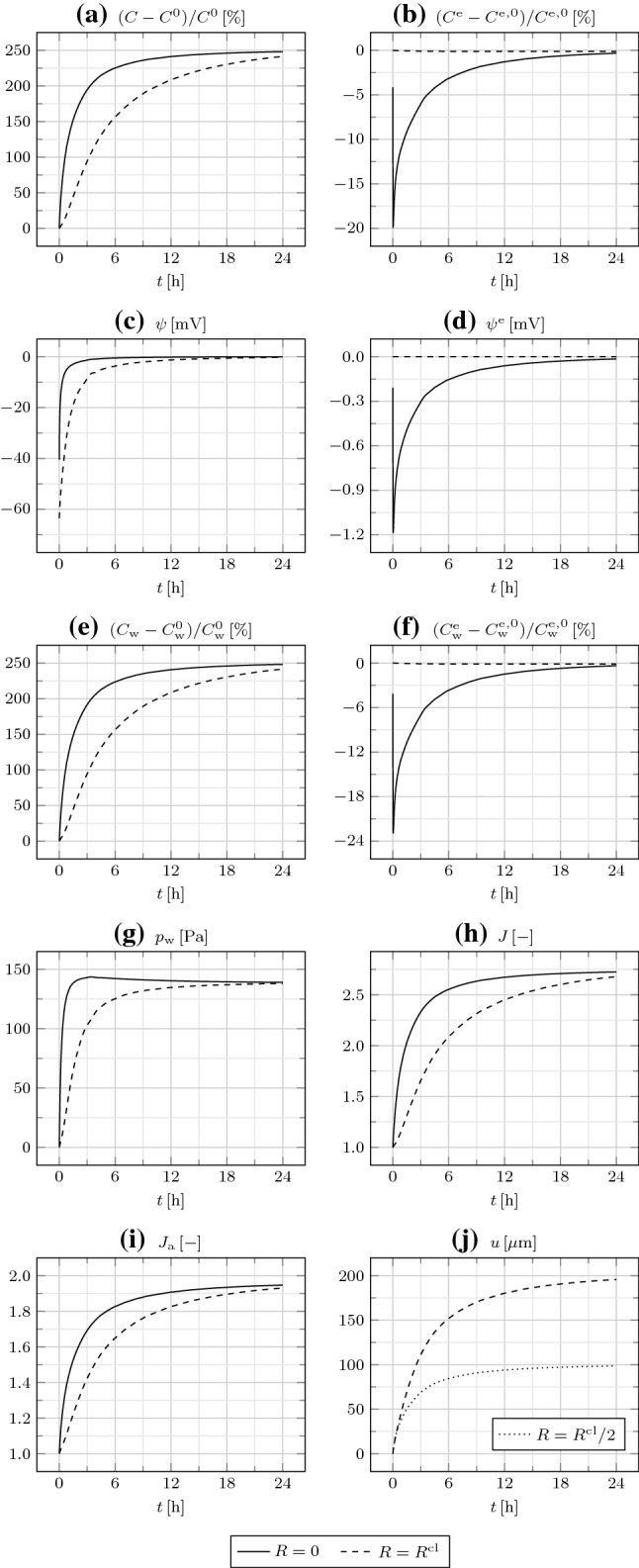
**a** Relative IC osmotic concentration $(C-C^0)/C^0$, **b** relative EC osmotic concentration $(C^{\mathrm {e}}-C^{\mathrm {e,0}})/C^{\mathrm {e,0}}$, **c** IC electric potential $\psi$, **d** EC electric potential $\psi ^{\mathrm {e}}$, **e** relative IC water concentration $(C_{\mathrm {w}}-C_{\mathrm {w}}^0)/C_{\mathrm {w}}^0$, **f** relative EC water concentration $(C_{\mathrm {w}}^{\mathrm {e}}-C_{\mathrm {w}}^\mathrm {e,0})/C_{\mathrm {w}}^{\mathrm {e,0}}$, **g** water pressure $p_{\mathrm {w}}$, **h** Jacobian *J*, and **i** areal Jacobian $J_{\mathrm {a}}$ at $R=0$ and $R=R^{\mathrm {cl}}$, and **j** radial displacement *u* at $R=R^{\mathrm {cl}}/2$ and $R=R^{\mathrm {cl}}$, as a function of time *t* in the presence of gap junctions

In Fig. 8, we investigate the cluster behavior in the presence of GJs until the steady state, reached at about $24\,{\mathrm {h}}$. The IC fields monotonically increase with time, both at $R=0$ and, albeit slower, as ions and water flow outward through GJs, at $R=R^{\mathrm {cl}}$. At the steady state, the IC fields attain the uniform values $(C-C^0)/C^0=(C_{\mathrm {w}}-C_{\mathrm {w}}^0)/C_{\mathrm {w}}^0\approx 2.5$ and $\psi =0$. The EC fields at $R=0$ rapidly decrease in the first $2\,{\mathrm {min}}$ and then slowly increase. At the steady state, the EC fields $C^{\mathrm {e}}-C^{\mathrm {e,0}}$, $C_{\mathrm {w}}^{\mathrm {e}}-C_{\mathrm {w}}^{\mathrm {e,0}}$, and $\psi ^{\mathrm {e}}$ attain uniform zero values. Therefore, the EC space is undeformed at the steady state. Notably, at the steady state $c=C/(v_{\mathrm {w}}C_{\mathrm {w}})=C^0=C^{\mathrm {e,0}}=C^{\mathrm {e}}/(v_{\mathrm {w}}C_{\mathrm {w}}^{\mathrm {e}})=c^{\mathrm {e}}$ [see Eq. (32)]; moreover, we could show that $c_{\mathrm {i}}=c_{\mathrm {i}}^{\mathrm {e}}=C_{\mathrm {i}}^{\mathrm {e,0}}\,\forall {\mathrm {i}}$. The volume ratio *J* behaves similar to $C_{\mathrm {w}}$, as $C_{\mathrm {w}}^{\mathrm {e}}$ is not significant for *J* in the absence of TJs. While initially $u(R^{\mathrm {cl}}/2)\approx u(R^{\mathrm {cl}})$, they progressively diverge and, at the steady state, $u(R^{\mathrm {cl}})=2u(R^{\mathrm {cl}}/2)\approx 0.4\,R^{\mathrm {cl}}$. To conclude, this simulation reveals that, in the absence of TJs and for a sufficiently compliant cluster [such that $p_{\mathrm {w}}$ is irrelevant in Eq. (40)] devoid of ion pumps, at the steady state the *current* IC and EC ion concentrations and the IC and EC electric potentials become equal to those of the bath surrounding the cluster, in turn coinciding with the initial EC ones. This is accompanied by large cluster deformations, exclusively attributable to the deformation of the IC space.

### Introducing tight junctions

**Fig. 9:**
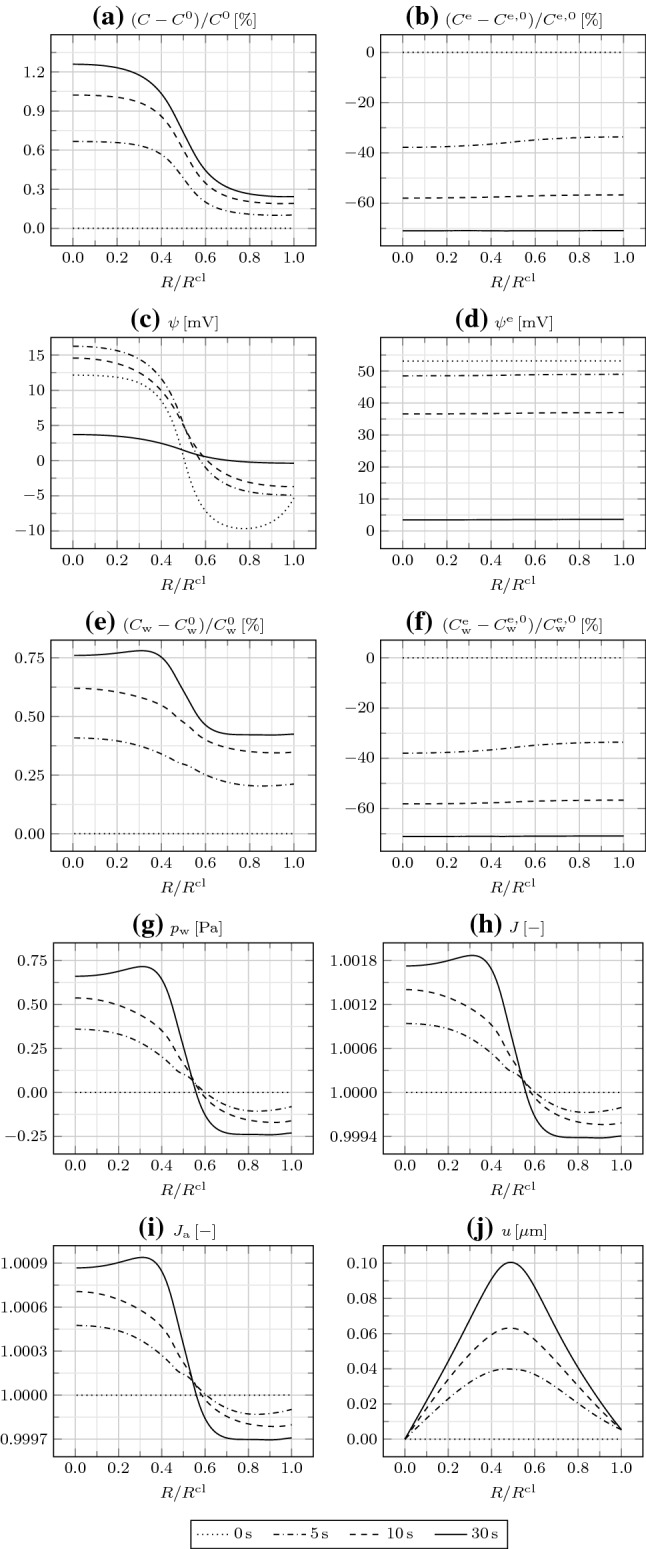
**a** Relative IC osmotic concentration $(C-C^0)/C^0$, **b** relative EC osmotic concentration $(C^{\mathrm {e}}-C^{\mathrm {e,0}})/C^{\mathrm {e,0}}$, **c** IC electric potential $\psi$, **d** EC electric potential $\psi ^{\mathrm {e}}$, **e** relative IC water concentration $(C_{\mathrm {w}}-C_{\mathrm {w}}^0)/C_{\mathrm {w}}^0$, **f** relative EC water concentration $(C_{\mathrm {w}}^{\mathrm {e}}-C_{\mathrm {w}}^\mathrm {e,0})/C_{\mathrm {w}}^{\mathrm {e,0}}$, **g** water pressure $p_{\mathrm {w}}$, **h** Jacobian *J*, **i** areal Jacobian $J_{\mathrm {a}}$, and **j** radial displacement *u* as a function of $R/R^{\mathrm {cl}}$ at different times, in the presence of gap and tight junctions

In this section, we comment on the cluster response in the presence of TJs, that is, by considering that the EC space cannot exchange neither water nor ions with the bath surrounding the cluster. Boundary conditions (9) and (12) now hold. As in Sect. 4.3, we also account for GJs. We display the results of the simulation in Fig. 9, by focusing on a relatively short time interval of $30\,{\mathrm {s}}$.

As for the previous case, the large $D_{\mathrm {{Na}}^{+}}^{\mathrm {m}}$ in $\varOmega _{\mathrm {in}}$ leads to a rapid inflow of ${\mathrm {Na}^+}$ from the EC to the IC space. However, in the presence of TJs, the ions of the outside bath cannot replace those lost by the EC space. Therefore, given that $\varPhi _0\gg \varPhi _0^{\mathrm {e}}$, the increase in *C* with time is limited, while the decrease in $C^{\mathrm {e}}$ is more pronounced and nearly uniform with *R*.

While $\psi ^{\mathrm {e}}$ remained nearly null everywhere in the absence of TJs, such that $\psi ^{\mathrm {m}}$ practically coincided with $\psi$, here both $\psi$ and $\psi ^{\mathrm {e}}$ contribute to $\psi ^{\mathrm {m}}$, being comparable in magnitude. We observe that, initially, $\psi ^{\mathrm {m}}$ is very close to the value registered in the absence of TJs. We further note that the positive $\psi ^{\mathrm {e}}$ at $R=R^{\mathrm {cl}}$ corresponds to the *transepithelial potential* established by TJs (Nuccitelli 2003).

Following ${\mathrm {Na}^+}$, water molecules pass from the EC to the IC space by osmosis through aquaporins, leading to an increase in $C_{\mathrm {w}}$ and to a decrease in $C_{\mathrm {w}}^{\mathrm {e}}$. As reported for *C* and $C^{\mathrm {e}}$, given the impermeability of the boundary to water and the large difference between $\varPhi _0$ and $\varPhi _0^{\mathrm {e}}$, $C_{\mathrm {w}}$ little increases, while $C_{\mathrm {w}}^{\mathrm {e}}$ strongly decreases.

Given the limited water redistribution, the mechanical fields are much smaller in magnitude than in the absence of TJs. Furthermore, we highlight that *J* and consequently $p_{\mathrm {w}}$ are negative in $\varOmega _{\mathrm {out}}$, meaning that the decrease in the EC volume is larger than the increase in the IC volume. Indeed, we remark that, in the presence of TJs, the great disparity between $|C_{\mathrm {w}}-C_{\mathrm {w}}^0|$ and $|C_{\mathrm {w}}^{\mathrm {e}}-C_{\mathrm {w}}^{\mathrm {e,0}}|$ makes both contributions important for the estimation of *J* through Eq. (30). The radial displacement *u* increases from $R=0$ to $R=R^{\mathrm {cl}}/2$, though remaining very small, and then decreases becoming nearly zero at $R=R^{\mathrm {cl}}$.

**Fig. 10:**
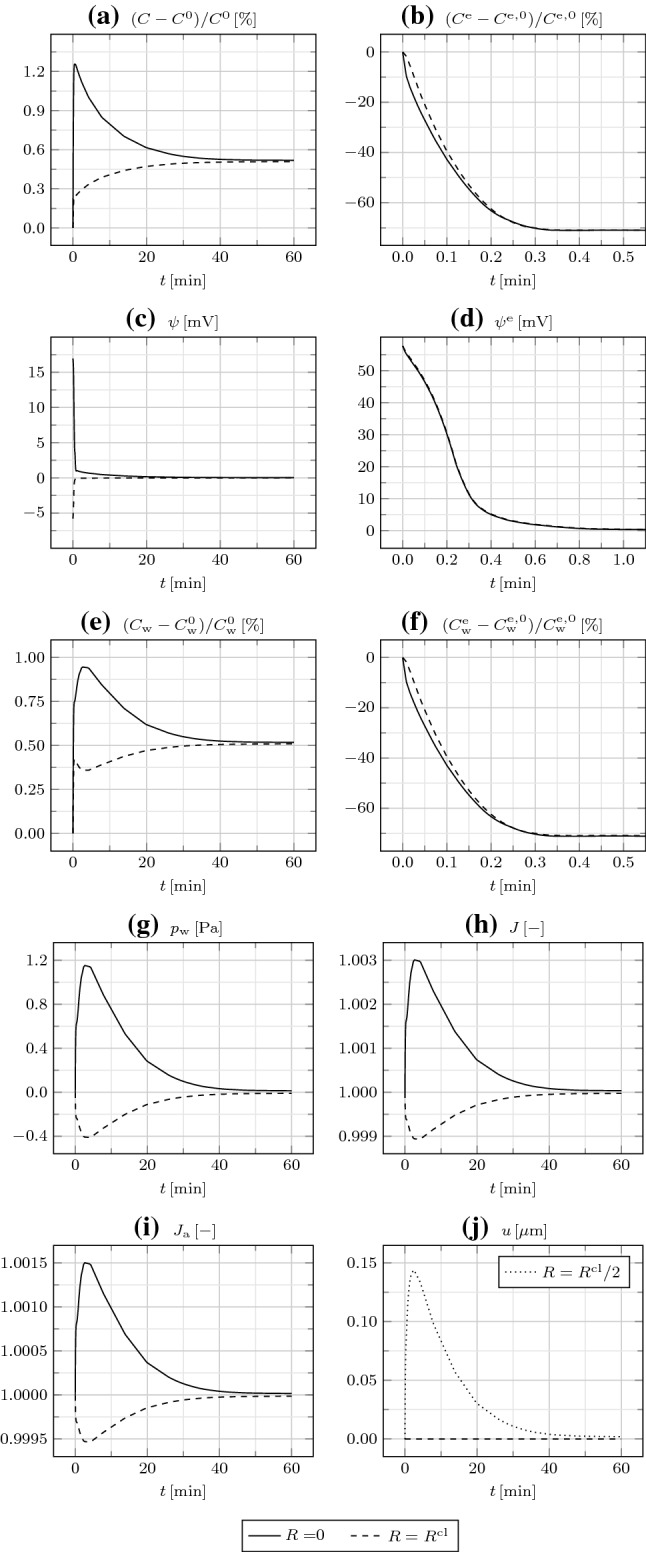
**a** Relative IC osmotic concentration $(C-C^0)/C^0$, **b** relative EC osmotic concentration $(C^{\mathrm {e}}-C^{\mathrm {e,0}})/C^{\mathrm {e,0}}$, **c** IC electric potential $\psi$, **d** EC electric potential $\psi ^{\mathrm {e}}$, **e** relative IC water concentration $(C_{\mathrm {w}}-C_{\mathrm {w}}^0)/C_{\mathrm {w}}^0$, **f** relative EC water concentration $(C_{\mathrm {w}}^{\mathrm {e}}-C_{\mathrm {w}}^{\mathrm {e,0}})/C_{\mathrm {w}}^{\mathrm {e,0}}$, **g** water pressure $p_{\mathrm {w}}$, **h** Jacobian *J*, and **i** areal Jacobian $J_{\mathrm {a}}$ at $R=0$ and $R=R^{\mathrm {cl}}$, and **j** radial displacement *u* at $R=R^{\mathrm {cl}}/2$ and $R=R^{\mathrm {cl}}$, as a function of time *t* in the presence of gap and tight junctions

In Fig. 10, we display the time evolution of the relevant fields until the steady state. After quickly increasing in the first $30\,{\mathrm {s}}$, $C(R=0)$ slowly decreases to a steady state value $\approx 1.005\,C^0$, reached at about $t=60\,{\mathrm {min}}$. Similarly, $C(R^{\mathrm {cl}})$ increases quite rapidly in the first $30\,{\mathrm {s}}$, but then keeps increasing, though slower, until the same steady state value of *C*(0). Both $C^{\mathrm {e}}(0)$ and $C^{\mathrm {e}}(R^{\mathrm {cl}})$ decrease to the same steady state value $\approx 0.3\,C^{\mathrm {e,0}}$ at about $t=30\,{\mathrm {s}}$. Therefore, we conclude that ${\mathrm {Na}^+}$ ions electro-diffuse from the EC to the IC space of $\varOmega _{\mathrm {in}}$ in the first $30\,{\mathrm {s}}$ and then flow from $\varOmega _{\mathrm {in}}$ to $\varOmega _{\mathrm {out}}$ through GJs until the steady state, when *C* becomes uniform within the whole cluster.

The evolution of $C_{\mathrm {w}}$ and $C_{\mathrm {w}}^{\mathrm {e}}$ at $R=0$ and $R=R^{\mathrm {cl}}$ in the first $30\,{\mathrm {s}}$ is analogous to that observed for *C* and $C^{\mathrm {e}}$. However, between approximately $t=30\,{\mathrm {s}}$ and $t=3\,{\mathrm {min}}$, $C_{\mathrm {w}}(0)$ increases, while $C_{\mathrm {w}}(R^{\mathrm {cl}})$ decreases. This suggests that, in this time interval, some water flows from $\varOmega _{\mathrm {out}}$ to $\varOmega _{\mathrm {in}}$, either directly through GJs or by passing through the EC space. After $3\,{\mathrm {min}}$, water starts flowing back from $\varOmega _{\mathrm {in}}$ to $\varOmega _{\mathrm {out}}$, until both $C_{\mathrm {w}}(0)$ and $C_{\mathrm {w}}(R^{\mathrm {cl}})$ reach the same steady state value $\approx 1.005\,C_{\mathrm {w}}^0$. Notably, $c=C^0=C^{\mathrm {e,0}}=c^{\mathrm {e}}$ at the steady state; moreover, we could show that $c_{\mathrm {i}}=c_{\mathrm {i}}^{\mathrm {e}}\approx C_{\mathrm {i}}^0\,\forall {\mathrm {i}}$.

The Jacobian *J* and the areal Jacobian $J_{\mathrm {a}}$ increase at $R=0$ and decrease at $R=R^{\mathrm {cl}}$ until $t=3\,{\mathrm {min}}$ and then asymptotically tend to one. In particular, $J_{\mathrm {a}}$ is equal to the out-of-plane stretch $J/J_{\mathrm {a}}$. The radial displacement *u* at $R=R^{\mathrm {cl}}/2$ increases until $t=3\,{\mathrm {min}}$ and then goes to zero.

To conclude, in the presence of TJs and in the absence of ion pumps, at the steady state the *current* IC and EC ion concentrations are equal and close to the initial IC values, and both the IC and EC spaces are electroneutral. Moreover, within the same material point, the volume increase in the IC space balances the volume decrease in the EC space, such that the cluster is globally undeformed.

## Concluding remarks

We have proposed a continuum finite strain theory for the coupling of electrostatics, ion transport, water transport, and mechanics of a closely packed cell cluster.

Specifically, we have regarded the cluster as the superposition of a solid network of cytoskeletal filaments and anchoring junctions and intracellular (IC) and extracellular (EC) solutions of water and ions. We have described the mechanics of the solid network through compressible hyperelasticity. Given the diluteness of the IC and EC solutions and the incompressibility of all the constituents, volumetric deformations are established by water redistribution only. We have obtained the IC and EC fluxes, the first being allowed by gap junctions, by considering cross-diffusing effects. Correspondingly, the IC and EC water fluxes result from the contributions of water pressure and electro-osmosis, while the IC and EC ion fluxes are due to electro-diffusion and convection. We have further accounted for transmembrane osmosis and ion electro-diffusion through aquaporins and ion channels, respectively.

We have tested our model to an in-plane circular cluster whose central region $\varOmega _{\mathrm {in}}$ presents an overexpression of sodium channels. The model correctly predicts the accumulation of ions and, consequently, of water, in the IC space of $\varOmega _{\mathrm {in}}$, and the resulting depolarization and in-plane expansion. The presence of gap junctions smooths out the steep gradients in all the relevant fields otherwise existing at the boundary of $\varOmega _{\mathrm {in}}$. The contribution of the pressure to the water flux becomes relevant in stiff plant cell clusters, while in deformable animal cell clusters the water flow is exclusively dictated by osmotic phenomena. In the absence of tight junctions, the ion and water redistribution may be severe, leading to large deformations; moreover, the membrane potential and the volumetric deformation are essentially established by the IC fields only, as the EC space remains nearly electroneutral and undeformed, except for the very initial transient. Differently, in the presence of cluster-sealing tight junctions, the ion and water redistribution is much more limited, resulting in small deformations, and both the IC and EC spaces contribute to setting the membrane potential and the volumetric deformation.

The model may be quite straightforwardly complemented with the addition of (i) the active transmembrane ion transport through ion pumps, (ii) the dependency of the diffusivity of ion channels and gap junctions on the membrane potential and tension (so as to account for voltage gating and mechanosensitivity), and (iii) genetic and biochemical dynamics required for specific applications, as already addressed in the literature (Pietak and Levin 2016, 2017; Leronni et al. 2020).

A major and cumbersome advancement would be the inclusion of growth into the model, toward exploring the interplay between mechanical and bioelectrical dynamics in development and regeneration. This could be achieved by introducing an inelastic (growth) deformation gradient, multiplying the elastic contribution and being modulated by the membrane potential, so as to relate growth and depolarization (Sundelacruz et al. 2009; Ambrosi et al. 2019; Silver et al. 2020).

## Data Availability

The finite element *Comsol Multiphysics*^®^  model has been run at the Applied Acoustics Lab—University of Brescia. The code is available upon request.

## Governing equations for the one-dimensional axisymmetric benchmark

In a 1D axisymmetric space dimension, equilibrium (1) reduces to

52 $$\begin{aligned} P_{rR}'+\frac{1}{R}(P_{rR}-P_{\theta \varTheta })=0\,, \end{aligned}$$

where $P_{rR}'=\partial P_{rR}/\partial R$, and $P_{rR}$ and $P_{\theta \varTheta }$ are the radial and circumferential nominal stresses, given by [see Eq. (35)]

53a $$\begin{aligned} P_{rR}&=G\left( F_{rR}-\frac{1}{F_{rR}}\right) +\lambda \frac{\ln J}{F_{rR}}-p_{\mathrm {w}}\frac{J}{F_{rR}}\nonumber \\&\quad +\frac{F_{rR}}{2\varepsilon _0\varepsilon _{\mathrm {r}}J}\left[ D^2+\left( D^{\mathrm {e}}\right) ^2\right] \,, \end{aligned}$$

53b $$\begin{aligned} P_{\theta \varTheta }&=G\left( F_{\theta \varTheta }-\frac{1}{F_{\theta \varTheta }}\right) +\lambda \frac{\ln J}{F_{\theta \varTheta }}-p_{\mathrm {w}}\frac{J}{F_{\theta \varTheta }}\nonumber \\&\quad -\frac{F_{rR}^2}{2\varepsilon _0\varepsilon _{\mathrm {r}}JF_{\theta \varTheta }}\left[ D^2+\left( D^{\mathrm {e}}\right) ^2\right] \,, \end{aligned}$$

where

54 $$\begin{aligned} J=F_{rR}F_{\theta \varTheta }F_{zZ}\,, \end{aligned}$$

with

55a $$\begin{aligned}&F_{rR}=1+u'\,, \end{aligned}$$

55b $$\begin{aligned}&F_{\theta \varTheta }=1+\frac{u}{R}\,, \end{aligned}$$

and $F_{zZ}$ denoting the radial, circumferential, and out-of-plane deformation gradient components (or stretches), and *u* being the radial displacement. Under plane stress conditions, the out-of-plane nominal stress $P_{zZ}$ is zero:

56 $$\begin{aligned} P_{zZ}&=G\left( F_{zZ}-\frac{1}{F_{zZ}}\right) +\lambda \frac{\ln J}{F_{zZ}}-p_{\mathrm {w}}\frac{J}{F_{zZ}}\nonumber \\&\quad -\frac{F_{rR}^2}{2\varepsilon _0\varepsilon _{\mathrm {r}}JF_{zZ}}\left[ D^2+\left( D^{\mathrm {e}}\right) ^2\right] =0\,. \end{aligned}$$

We equip Eq. (52) with the following boundary conditions [see Eq. (5)], with that in $R=0$ ensuing from symmetry:

57 $$\begin{aligned} u(0)=0\,,\quad P_{rR}(R^{\mathrm {cl}})=0\,. \end{aligned}$$

Mass balances (2) and (3) become

58a $$\begin{aligned}&\varPhi _0{\dot{C}}_{\mathrm {w}}+J_{\mathrm {w}}'+\frac{J_{\mathrm {w}}}{R}=-J_{\mathrm {w}}^{\mathrm {m}}\frac{2}{R^{\mathrm {c}}}\,, \end{aligned}$$

58b $$\begin{aligned}&\varPhi _0^{\mathrm {e}}{\dot{C}}_{\mathrm {w}}^{\mathrm {e}}+(J_{\mathrm {w}}^{\mathrm {e}})'+\frac{J_{\mathrm {w}}^{\mathrm {e}}}{R}=J_{\mathrm {w}}^{\mathrm {m}}\frac{2}{R^{\mathrm {c}}}\,, \end{aligned}$$

58c $$\begin{aligned}&\varPhi _0{\dot{C}}_{\mathrm {i}}+J_{\mathrm {i}}'+\frac{J_{\mathrm {i}}}{R}=-J_{\mathrm {i}}^{\mathrm {m}}\frac{2}{R^{\mathrm {c}}}\,, \end{aligned}$$

58d $$\begin{aligned}&\varPhi _0^{\mathrm {e}}{\dot{C}}_{\mathrm {i}}^{\mathrm {e}}+(J_{\mathrm {i}}^{\mathrm {e}})'+\frac{J_{\mathrm {i}}^{\mathrm {e}}}{R}=J_{\mathrm {i}}^{\mathrm {m}}\frac{2}{R^{\mathrm {c}}}\,, \end{aligned}$$

where the radial IC and EC nominal fluxes read [see Eq. (49)]

59a $$\begin{aligned} J_{\mathrm {w}}=-\frac{D_{\mathrm {w}}}{F_{rR}^2}\Biggl (\frac{v_{\mathrm {w}}C_{\mathrm {w}}}{{\mathcal {R}}T}p_{\mathrm {w}}'+\frac{F}{{\mathcal {R}}T}\sum _{\mathrm {i}}z_{\mathrm {i}}C_{\mathrm {i}}\psi ' +\frac{\sum _{\mathrm {{j}}\ne {\mathrm{i}}}C_\mathrm {j}}{C_{\mathrm {w}}}C_{\mathrm {w}}'\Biggr )\,, \end{aligned}$$

59b $$\begin{aligned} J_{\mathrm {w}}^{\mathrm {e}}=-\frac{D_{\mathrm {w}}^{\mathrm {e}}}{F_{rR}^2}\Biggl (\frac{v_{\mathrm {w}}C_{\mathrm {w}}^{\mathrm {e}}}{{\mathcal {R}}T}p_{\mathrm {w}}'+\frac{F}{{\mathcal {R}}T}\sum _{\mathrm {i}}z_{\mathrm {i}}C_{\mathrm {i}}^{\mathrm {e}}(\psi ^{\mathrm {e}})' \nonumber \\\quad +\frac{\sum _{\mathrm {{j}}\ne {\mathrm{i}}}C_\mathrm {j}^{\mathrm {e}}}{C_{\mathrm {w}}^{\mathrm {e}}}(C_{\mathrm {w}}^{\mathrm {e}})'\Biggr )\,, \end{aligned}$$

59c $$\begin{aligned} J_{\mathrm {i}}&=\frac{C_{\mathrm {i}}}{C_{\mathrm {w}}}J_{\mathrm {w}}-\frac{D_{\mathrm {i}}}{F_{rR}^2}\left( C_{\mathrm {i}}'-\frac{C_{\mathrm {i}}}{C_{\mathrm {w}}}C_{\mathrm {w}}'+\frac{F}{{\mathcal {R}}T}z_{\mathrm {i}}C_{\mathrm {i}}\psi '\right) \,, \end{aligned}$$

59d $$\begin{aligned} J_{\mathrm {i}}^{\mathrm {e}}&=\frac{C_{\mathrm {i}}^{\mathrm {e}}}{C_{\mathrm {w}}^{\mathrm {e}}}J_{\mathrm {w}}^{\mathrm {e}}-\frac{D_{\mathrm {i}}^{\mathrm {e}}}{F_{rR}^2}\left( (C_{\mathrm {i}}^{\mathrm {e}})'-\frac{C_{\mathrm {i}}^{\mathrm {e}}}{C_{\mathrm {w}}^{\mathrm {e}}}(C_{\mathrm {w}}^{\mathrm {e}})'+\frac{F}{{\mathcal {R}}T}z_{\mathrm {i}}C_{\mathrm {i}}^{\mathrm {e}}(\psi ^{\mathrm {e}})'\right) \,. \end{aligned}$$

The IC nominal concentration of water $C_{\mathrm {w}}$ and the transmembrane nominal fluxes $J_{\mathrm {w}}^{\mathrm {m}}$ and $J_{\mathrm {i}}^{\mathrm {m}}$ are still given by Eqs. (34) and (50). We supply Eqs. (58) with the following initial conditions [see Eqs. (6b), (31), and (41)]:

60a $$\begin{aligned}&p_{\mathrm {w}}=0\,,\quad C_{\mathrm {w}}^{\mathrm {e}}=\frac{1}{v_{\mathrm {w}}}\,, \end{aligned}$$

60b $$\begin{aligned}&C_{\mathrm {i}}=C_{\mathrm {i}}^0\,,\quad C_{\mathrm {i}}^{\mathrm {e}}=C_{\mathrm {i}}^\mathrm {e,0}\,, \end{aligned}$$

and with the following boundary conditions [see Eqs. (7), (9), (40), and (43)]:

61a $$\begin{aligned}&J_{\mathrm {w}}(0)=J_{\mathrm {w}}(R^{\mathrm {cl}})=0\,, \end{aligned}$$

61b $$\begin{aligned}&J_{\mathrm {w}}^{\mathrm {e}}(0)=0\,, \nonumber \\&C_{\mathrm {w}}^{\mathrm {e}}(R^{\mathrm {cl}})=\frac{{\mathcal {R}}TC^{\mathrm {e}}(R^{\mathrm {cl}})}{v_{\mathrm {w}}\left[ {\mathcal {R}}TC^\mathrm {e,0}+p_{\mathrm {w}}(R^{\mathrm {cl}})\right] }\quad \mathrm {without\,\,TJs}\,, \nonumber \\&J_{\mathrm {w}}^{\mathrm {e}}(R^{\mathrm {cl}})=0\quad \mathrm {with\,\,TJs}\,, \end{aligned}$$

61c $$\begin{aligned}&J_{\mathrm {i}}(0)=J_{\mathrm {i}}(R^{\mathrm {cl}})=0\,, \end{aligned}$$

61d $$\begin{aligned}&J_{\mathrm {i}}^{\mathrm {e}}(0)=0\,, \nonumber \\&C_{\mathrm {i}}^{\mathrm {e}}(R^{\mathrm {cl}})=v_{\mathrm {w}}C_{\mathrm {i}}^\mathrm {e,0}C_{\mathrm {w}}^{\mathrm {e}}(R^{\mathrm {cl}})\quad \mathrm {without\,\,TJs}\,, \nonumber \\&J_{\mathrm {i}}^{\mathrm {e}}(R^{\mathrm {cl}})=0\quad \mathrm {with\,\,TJs}\,. \end{aligned}$$

Finally, Gauss laws (4) reduce to

62a $$\begin{aligned}&D'+\frac{D}{R}=\varPhi _0F\sum _{\mathrm {i}}z_{\mathrm {i}}C_{\mathrm {i}}\,, \end{aligned}$$

62b $$\begin{aligned}&(D^{\mathrm {e}})'+\frac{D^{\mathrm {e}}}{R}=\varPhi _0^{\mathrm {e}}F\sum _{\mathrm {i}}z_{\mathrm {i}}C_{\mathrm {i}}^{\mathrm {e}}\,, \end{aligned}$$

where the IC and EC electric displacements are given by [see Eq. (44)]

63a $$\begin{aligned}&D=-\varepsilon _0\varepsilon _{\mathrm {r}}\frac{J}{F_{rR}^2}\psi '\,, \end{aligned}$$

63b $$\begin{aligned}&D^{\mathrm {e}}=-\varepsilon _0\varepsilon _{\mathrm {r}}\frac{J}{F_{rR}^2}(\psi ^{\mathrm {e}})'\,. \end{aligned}$$

We complement Eqs. (62) with the following boundary conditions [see Eqs. (10)–(12) and (45)]:

64a $$\begin{aligned}&D(0)=0\,,\quad D(R^{\mathrm {cl}})=\varepsilon _0\varepsilon _{\mathrm {r}}^{\mathrm {m}}\frac{\psi }{T^{\mathrm {m}}}\,, \end{aligned}$$

64b $$\begin{aligned}&D^{\mathrm {e}}(0)=0\,, \nonumber \\&\psi ^{\mathrm {e}}(R^{\mathrm {cl}})=0\quad \mathrm {without\,\,TJs}\,, \nonumber \\&D^{\mathrm {e}}(R^{\mathrm {cl}})=\varepsilon _0\varepsilon _{\mathrm {r}}^{\mathrm {tj}}\frac{\psi ^{\mathrm {e}}}{T^{\mathrm {tj}}}\quad {\mathrm {with\,\,TJs}}\,. \end{aligned}$$

## Finite element implementation

We solve the coupled governing equations presented in “Appendix A” by employing the finite element commercial software *Comsol Multiphysics*^®^. We use the General Form PDE interface to implement equilibrium (52), mass balances (58), and Gauss laws (62), and the Domain ODEs and DAEs interface to impose the plane stress constraint (56). We employ quadratic Lagrange shape functions to approximate the independent variables *u*, $p_{\mathrm {w}}$, $C_{\mathrm {w}}^{\mathrm {e}}$, $C_{\mathrm {i}}$, $C_{\mathrm {i}}^{\mathrm {e}}$, $\psi$, $\psi ^{\mathrm {e}}$, and $F_{zZ}$. The mesh consists of 100 elements, whose size decreases linearly from the boundaries to the interface between $\varOmega _{\mathrm {in}}$ and $\varOmega _{\mathrm {out}}$, where the independent variables undergo steep gradients, especially in the absence of GJs. Specifically, the ratio between the largest and smallest elements is 10. We employ the BDF method for the time integration and adopt the Fully Coupled approach, equipped with the MUMPS linear solver, to solve all the discretized equations simultaneously at each time step.
